# Characterization and prognostic impact of ACTBL2-positive tumor-infiltrating leukocytes in epithelial ovarian cancer

**DOI:** 10.1038/s41598-023-49286-9

**Published:** 2023-12-18

**Authors:** N. E. Topalov, D. Mayr, C. Kuhn, A. Leutbecher, C. Scherer, F. B. T. Kraus, C. V. Tauber, S. Beyer, S. Meister, A. Hester, T. Kolben, A. Burges, S. Mahner, F. Trillsch, M. Kessler, U. Jeschke, B. Czogalla

**Affiliations:** 1 Department of Obstetrics and Gynecology, University Hospital, LMU Munich, Munich, Germany; 2 Institute of Pathology, Faculty of Medicine, University Hospital, LMU Munich, Munich, Germany; 3 Department of Obstetrics and Gynecology, University Hospital Augsburg, Augsburg, Germany; 4 Laboratory for Translational Cancer Immunology, LMU Gene Center, Munich, Germany; 5 Department of Medicine III, University Hospital, LMU Munich, Munich, Germany; 6 Department of Medicine I, University Hospital, LMU Munich, Munich, Germany

**Keywords:** Prognostic markers, Ovarian cancer, Oncogenesis, Actin, Cell migration

## Abstract

Actin beta-like 2 (ACTBL2) was recently identified as a new mediator of migration in ovarian cancer cells. Yet, its impact on tumor-infiltrating and thus migrating leukocytes (TILs) remains to date unknown. This study characterizes the subset of ACTBL2-expressing TILs in epithelial ovarian cancer (EOC) and elucidates their prognostic influence on the overall survival of EOC patients with special regard to different histological subtypes. Comprehensive immunohistochemical analyses of Tissue-Microarrays of 156 ovarian cancer patients revealed, that a tumor infiltration by ACTBL2-positive leukocytes was significantly associated with an improved overall survival (OS) (61.2 vs. 34.4 months; *p* = 0.006) and was identified as an independent prognostic factor (HR = 0.556; *p* = 0.038). This significant survival benefit was particularly evident in patients with low-grade serous carcinoma (OS: median not reached vs. 15.6 months, *p* < 0.001; HR = 0.058, *p* = 0.018). In the present cohort, ACTBL2-positive TILs were mainly composed of CD44-positive cytotoxic T-cells (CD8+) and macrophages (CD68+), as depicted by double-immunofluorescence and various immunohistochemical serial staining. Our results provide significant evidence of the prognostic impact and cellular composition of ACTBL2-expressing TILs in EOC. Complementary studies are required to analyze the underlying molecular mechanisms of ACTBL2 as a marker for activated migrating leukocytes and to further characterize its immunological impact on ovarian carcinogenesis**.**

## Introduction

Epithelial ovarian cancer (EOC) remains the fifth leading cause of cancer death in women and the most lethal tumor entity among gynecological cancer patients^1^. As a result of insufficient screening methods and a comparably late onset of primarily unspecific symptoms, EOC is mostly detected in an advanced stage with a consequently poor five-year survival rate of 47%^2^. Apart from the FIGO stage at initial diagnosis, decisive prognostic factors for overall survival include patient’s age, histological subtype, tumor grade and the volume of residual disease after primary debulking surgery as the most significant ones^3–6^. Although recent studies stated substantial differences in ovarian carcinogenesis regarding underlying molecular pathways and clinicopathological features^7–9^, first-line therapy of EOC to date still consists of cytoreductive surgery followed by adjuvant platinum-based chemotherapy, irrespective of distinct histological subtypes^10^. Maintenance treatment contains the use of VEGF-inhibitor bevacizumab^11,12^ and/or poly-ADP-ribose-polymerase inhibitors (PARPi), depending on the patient’s prior response to chemotherapy and the individual BRCA1/2 mutation and homologous recombination deficiency (HRD) status^13,14^. In contrast to other gynecological malignancies such as cervical and endometrial carcinoma, ovarian cancer shows the least susceptibility to immune therapy due to its comparably low tumor mutational burden (TMB)^15–18^. Despite several attempts to establish checkpoint inhibitors as a promising new option in EOC treatment, no significant prognostic benefit could be shown thus far^19,20^. Further development of targeted therapeutical approaches requires a definition of predictive factors for the response to immunotherapy and an increasingly better understanding of the distinct tumor biology and microenvironment.

Actin beta-like 2 (ACTBL2) is considered a newly discovered non-muscle actin isoform with 92% structural similarity to ß-actin^21,22^. Yet, recent studies corroborated a genetic distance from the six commonly known isoforms, with *ACTBL2* showing the highest number of non-conserved amino acid substitutions in comparative phylogenetic analyses^23^. Functional examinations in human melanoma cells revealed an interaction between ACTBL2 and gelsolin in the course of cellular lamellipodia formation^24^. Subsequent studies emphasized its significant motility-enhancing effect since a lack of ACTBL2 was associated with impaired cellular invasion abilities and an altered actin cytoskeleton structure^23^. Consistent with that, gene silencing of *ACTBL2* and its transcription factor Nuclear factor of activated T-cells 5 (*NFAT5*) resulted in decreased migration of biomechanically activated vascular smooth muscle cells (VSMC)^25^. Besides a significant upregulation in colorectal and hepatocellular carcinoma, *ACTBL2* was additionally identified as a potential risk gene in ovarian cancer^26–28^. Comprehensive analyses confirmed a statistically independent prognostic disadvantage for EOC patients with impaired overall survival upon positive ACTBL2-expression in the according tumor cells^29^. Further, functional assays after targeted gene silencing proved its significant modulating impact on proliferation and especially migration of high-grade serous ovarian carcinoma cells^29^. Despite growing evidence on its crucial and fundamental impact on cellular motility, the extent to which ACTBL2 is expressed in tumor-infiltrating and thus migrating leukocytes (TILs) remains to date unknown.

The present study aims at identifying and characterizing the subset of ACTBL2-expressing TILs in epithelial ovarian cancer and at elucidating their prognostic influence on the overall survival of EOC patients with special regard to different histological subtypes.

## Methods

### Ethical approval

The present study was carried out according to the guidelines of the Ethics Committee of the Ludwig-Maximilians-University (LMU), Munich, Germany (approval number 227-09, 18-392 and 19-972). All tissue samples utilized were derived from material, which was primarily used for histopathological assessment and stored in the archives of our Department of Obstetrics and Gynecology, LMU, Munich, Germany. Diagnostic procedures on the tumor tissue were completed before its scientific use, securing a full anonymization of the patients’ data during all experimental and analytical stages. All experiments were performed in strict compliance with the standards of the Declaration of Helsinki (1975), given the written informed consent of all patients/participants.

### Patients and specimens

Tissue samples of 156 patients who underwent cytoreductive surgery for EOC between 1990 and 2002 at the Department of Obstetrics and Gynecology, Ludwig-Maximilians-University in Munich were collected, with distinct biopsies of representative tumor areas being combined in a Tissue-Microarray (TMA) by the Department of Pathology, LMU, Munich. The corresponding clinical data was gained from the patients’ charts and the consecutive follow-up data was provided by the Munich Cancer Registry (MCR). None of the patients has had neoadjuvant chemotherapy in the clinical course and only patients with pathologically confirmed EOC were included in the collective. Patients with benign, precursor or borderline lesions were accordingly excluded from the study.

All samples were formalin-fixed and paraffin-embedded (FFPE) prior to being assessed by specialized gynecological pathologists regarding histopathological criteria. The specimens were classified into the four most common histological subtypes [serous (n = 110), clear cell (n = 12), endometrioid (n = 21) and mucinous (n = 13) carcinoma; Table 1] and consecutively graded respecting the currently valid WHO classification^30^. Serous ovarian cancer tissue was subdivided into low and high grading. Samples of endometrioid histology were graded from G1 to G3 as well as tissue from mucinous carcinoma since this subtype is lacking explicit WHO classification criteria. Clear cell cancer was always categorized as G3. Tumor staging was executed in line with the FIGO classification [I (n = 35), II (n = 10), III (n = 103), IV (n = 3)] based on available data on primary tumor extension (n = 155) according to the TNM classification as a globally recognized standard for the primary tumor site and size (T), regional lymph node involvement (N) and the presence of distant metastases (M)^31^. Regarding lymph node involvement, data was accessible in 95 cases [N0 (n = 43), N1 (n = 52)], whereas data on distant metastasis by the time of cytoreductive surgery was only obtainable in 9 cases [M0 (n = 3), M1 (n = 6)]. Information on the FIGO stage and histological grading are unavailable in 5 and 12 cases, respectively.

**Table 1:** Clinicopathological features of 156 ovarian cancer patients included in this study.

Clinicopathological parameters	n	Percentage (%)
Histology		
Serous	110	70.5
Clear cell	12	7.7
Endometrioid	21	13.5
Mucinous	13	8.3
Primary tumor extension		
TX	1	0.6
T1	40	25.6
T2	18	11.5
T3	97	62.3
Nodal status		
pNX	61	39.1
pN0	43	27.6
pN1	52	33.3
Distant metastasis		
pMX	147	94.2
pM0	3	1.9
pM1	6	3.8
Grading serous		
Low	24	21.8
High	80	72.7
Grading endometrioid		
G1	6	28.6
G2	5	23.8
G3	8	38.1
Grading mucinous		
G1	6	46.2
G2	6	46.2
G3	0	0
Grading clear cell		
G3	12	100.0
FIGO stage		
I	35	22.4
II	10	6.4
III	103	66.0
IV	3	1.9
Patients’ age		
≤ 60 years	83	53.2
> 60 years	73	46.8

### Serial tissue slides

For a distinct characterization of particular cells by comparative immunohistochemical analyses, serial slides of selected patients’ tissue of each histological subtype from the previously described collective were produced. Formalin-fixed and paraffin-embedded (FFPE) ovarian cancer tissue was cut into at least four successive, 3 µm thick slices using a sledge-microtome (Hn 40, Reichert-Jung, Germany). After stretching the slices in a water bath, the tissue was placed on ascending numbered microscope slides (Menzel-Gläser Superfrost Plus, ThermoScientific, Gerhard Menzel GmbH, Braunschweig) and was dried for 12 h in an incubator at 50 °C. Serial tissue slides from primary fallopian tube cancer were provided by the Department of Pathology, LMU, Munich.

### Immunohistochemistry

Immunohistochemical staining was conducted as previously described^29^. Formalin-fixed and paraffin-embedded tissue slides were dewaxed in Roticlear (Roth, Karlsruhe, Germany) for 20 min and then shortly washed in 100% ethanol. After blocking the endogenous peroxidase by using 3% H_2_O_2_ in methanol for 20 min, the samples were gradually rehydrated in descending ethanol concentrations (100%, 70% and 50%) before being put in distilled water. In a next step, the slides were placed in a pressure cooker containing a boiling sodium citrate buffer (0.1 M citric acid, 0.1 M sodium citrate; pH = 6) and were followingly heated for 5 min. After cooling, the specimens were washed again in distilled water and afterwards twice in phosphate-buffered saline (PBS) for 2 min each. Intending to avoid an unspecific staining reaction, a blocking solution [Reagent 1; ZytoChem Plus HRP Polymer System (mouse/rabbit); Zytomed, Berlin, Germany] was applied on the tissue for 5 min at room temperature (RT) prior to the incubation with distinct primary antibodies under reagent-specific conditions as listed in detail in the Supplementary file, Table S1. Following that, the slides were washed twice in PBS and subsequently treated with a post-block solution [Reagent 2; ZytoChem Plus HRP Polymer System (mouse/rabbit); Zytomed, Berlin, Germany] for 20 min at RT before another 30 min incubation with an HRP-polymer, containing bound anti-mouse and anti-rabbit antibodies [Reagent 3; ZytoChem Plus HRP Polymer System (mouse/rabbit); Zytomed, Berlin, Germany]. The staining was visualized by applying 3,3′diaminobenzidine (DAB) and the according substrate buffer (Liquid DAB and Substrate Chromogen System; DAKO, Munich, Germany) on the tissue. After washing the slides in distilled water to end the reaction, Mayer’s acidic hemalum (Waldeck, Münster, Germany) was used for counterstaining. Next, the tissue was dehydrated in a series of ethanol with rising concentrations (70%, 96% and 100%) before being put in Roticlear and subsequently being covered. Kidney, placenta, colon and tonsil tissue served as negative and positive controls to determine the most suitable antibody dilution and to prove the specificity of the immunoreaction (Figure S1). Regarding the negative controls, each primary antibody was replaced by a species-specific isotype control antibody (BioGenex, Fremont, CA, USA).

### Immunofluorescence

For immunofluorescence staining, the FFPE slides were pre-treated as previously described for immunohistochemistry. In order to prevent an unspecific binding of the primary antibodies, a blocking solution (Ultra Vision Protein Block; ThermoScientific, Lab Vision, Fremont, CA, USA) was applied on the tissue for 15 min at RT. After gently removing the surplus of blocking solution, the slides were incubated with a mixed solution of primary antibodies against ACTBL2 and CD45, respectively CD44, for 16 h at 4 °C (for detailed information on all antibodies used, see Supplementary file, Table S1). Next, the slides were washed twice in PBS and treated with fluorophore-labelled and species-specific secondary antibodies (Table S2) in the dark for 30 min at RT. Following, the specimens were once again washed twice in PBS prior to being covered in a dry state with mounting medium containing DAPI for nuclear counterstaining [Vectashield Anti-fade mounting medium with DAPI (H-1200); Vector Laboratories, Burlingame, CA, USA]. The double-staining was observed by using a confocal laser microscope (Axiophot fluorescence microscope; Zeiss, Oberkochen, Germany) and subsequently analyzed with the corresponding software AxioVision.

### Antibody specificity and validation

For proving the specificity of the used anti-ACTBL2 antibody, in vitro experiments upon small interfering RNA (siRNA) knockdown of *ACTBL2* were performed as previously described^29^. UWB1.289 cells (serous ovarian cancer, BRCA1 negative—ATCC, Rockville, MD, USA) were seeded on sterile 6-well plates and maintained in culture using RPMI 1640 GlutaMAX Medium (Gibco, Paisley, UK) supplemented with 10% fetal bovine serum (FBS; Gibco, Paisley, UK) in a humified incubator at 37 °C and 5% CO_2_ until reaching a cell density of 70%. Afterwards, transfection was executed by using siRNA for *ACTBL2* (GeneSolution siRNA, Qiagen Sciences, MD, USA; for detailed information on corresponding sequences, see Figure S2 g) and Lipofectamine RNAiMAX reagent (Invitrogen, Carlsbad, CA, USA) according to the manufacturer’s protocol in OptiMEM Reduced Serum Medium (ThermoFisher Scientific, Waltham, MA, USA). After 48 h of incubation under the above mentioned conditions, the cells were harvested and used for mRNA isolation using the RNeasy Mini Kit (Qiagen, Venlo, Netherlands). 1 µg RNA was utilized for conversion into cDNA by utilizing the MMLV Reverse Transcriptase 1st-strand cDNA Synthesis Kit (Epicentre, Madison, WI, USA). mRNA expression of *ACTBL2* after siRNA knockdown was quantified by qPCR using FastStart Essential DNA Probes Master and gene-specific primers (Roche, Basel, Switzerland, Figure S2 g). The relative expression was calculated for each sequence by the 2^−∆∆Ct^ formula^32^ using *GAPDH* and *ß-actin* as housekeeping genes. Figure S2 a shows the successful and significant downregulation of *ACTBL2* up to 90% (sequence 3). Each siRNA knockdown and qPCR were repeated three times.

In order to prove a concordant decrease of ACTBL2 on a protein level, immunocytochemical (ICC) staining after 48 h of *ACTBL2* knockdown in UWB1.289 cells was executed. 5 × 10^4^ UWB1.289 cells were seeded per well in sterile 4-well chamber slides (Lab-Tek II Chamber Slides, ThermoFisher Scientific, Denmark) and maintained in culture overnight before executing siRNA knockdown of *ACTBL2* as previously described. Next, the slides were washed twice for 5 min with PBS before being fixed in 100% methanol and ethanol (1:1) at room temperature for 15 min. After drying, the slides were treated with goat-derived serum (Vectastain Elite rabbit-IgG-kit, Vector Laboratories, Burlingame, CA, USA) for 20 min at room temperature to avoid unspecific background staining. After another washing step, the slides were incubated with the aforementioned primary anti-ACTBL2 antibody (Table S1) in a 1:400 dilution for 16 h at 4 °C overnight. Following, a biotinylated secondary anti-rabbit antibody (Vectastain Elite rabbit-IgG-kit, Vector Laboratories, Burlingame, CA, USA) was applied for 30 min before subsequently treating the slides with an avidin–biotin-peroxidase complex (Vectastain Elite rabbit-IgG-kit, Vector Laboratories, Burlingame, CA, USA) for another 30 min. The staining was visualized by using chromogen 3-amino-9-ethylcarbazole (AEC+, DAKO, Hamburg, Germany) for 10 min with the reaction being stopped by placing the slides in distilled water. Counterstaining was executed with Mayer’s acidic hemalum (Waldeck, Münster, Germany) before finally covering the slides using an aqueous mounting medium (Aquatex, Merck, Darmstadt, Germany). The results obtained are shown in Figure S2 b–f, displaying a significant decrease in intracellular ACTBL2 expression.

### Staining evaluation and statistical analysis

Specimens of all 156 EOC patients were analyzed after the successfully performed immunohistochemical staining of ACTBL2 with special regard to the presence of ACTBL2-expressing TILs by using a Leitz photomicroscope (Wetzlar, Germany). Patients were divided binarily into two groups (0 = ACTBL2-positive TILs *not* detectable, 1 = ACTBL2-positive TILs detectable), thus enabling subsequent statistical analyses using IBM SPSS Statistics 28.0 (IBM Corporation, Armonk, NY, USA). Spearman’s analysis^33^ was executed to calculate bivariate correlations between pre-existing clinicopathological data and the current staining results. The overall survival was visualized by Kaplan–Meier estimates with the log-rank testing being used to check for statistical significance^34^. Appropriate cut-off values were primarily selected by performing a ROC curve analysis, being a reliable and widely recognized method for cut-off point definition^35^. Additionally, the Youden index was used to optimize the cut-off values by maximizing the sum of sensitivity and specificity^36,37^. For multivariate analyses, a Cox regression model considering clinicopathological characteristics and the investigated parameters was established^38^. For all analyses performed, p-values ≤ 0.05 were considered statistically significant.

## Results

### Tumor infiltration by ACTBL2-positive leukocytes is significantly associated with improved overall survival of ovarian cancer patients

**Figure 1:**
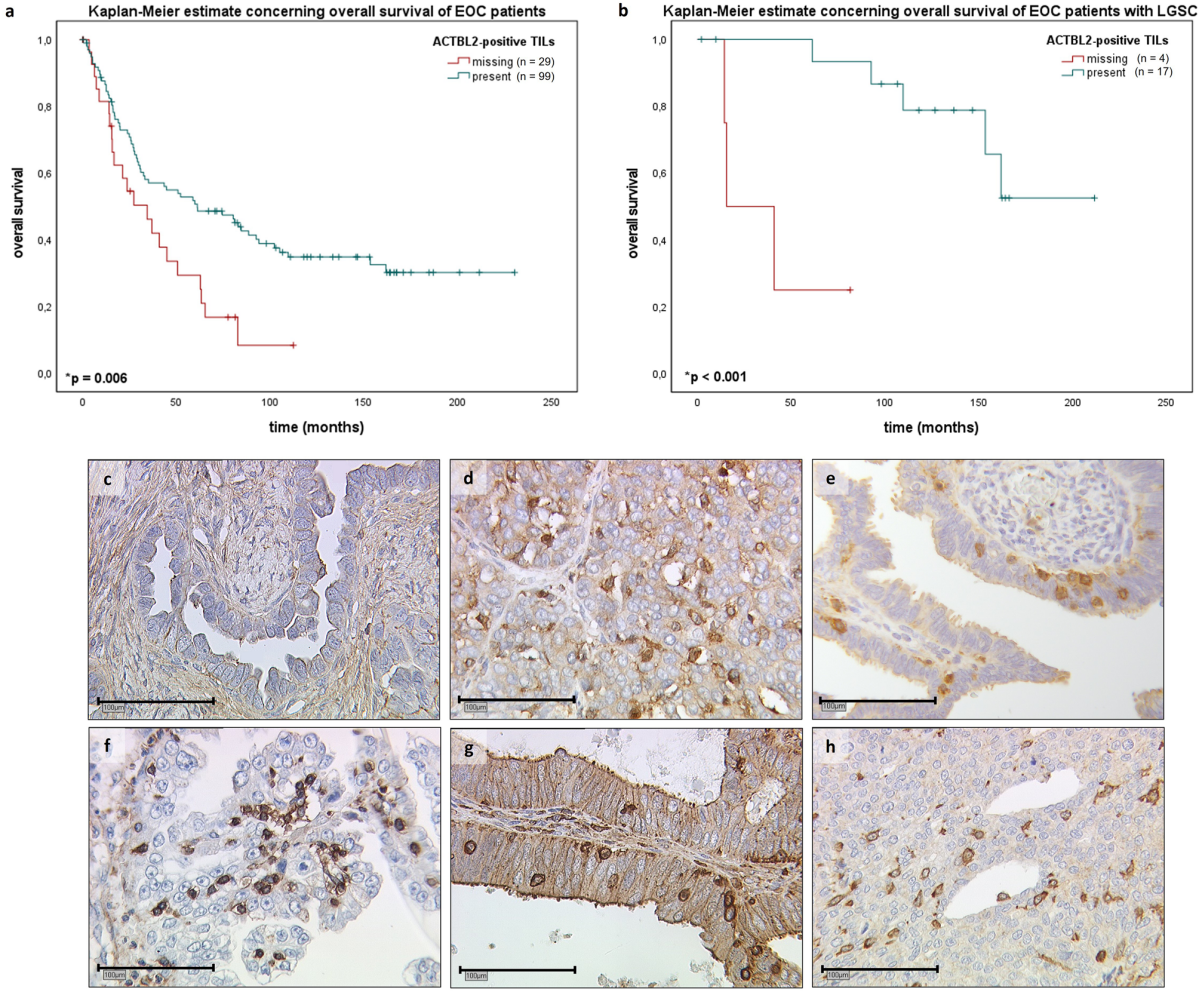
Kaplan–Meier estimates and exemplary photographs of tumor infiltration by ACTBL2-positive leukocytes as detected by immunohistochemistry.** (a)** Kaplan–Meier estimate (log-rank testing) considering the presence of ACTBL2-positive TILs in the overall collective of 156 EOC patients, being associated with a significantly longer overall survival (61.2 vs. 34.4 months; *p* = 0.006). **(b)** Kaplan–Meier estimate (log-rank testing) regarding the occurrence of ACTBL2-expressing TILs in low-grade serous ovarian cancer tissue, showing a significant survival benefit of the corresponding patients (median not reached vs. 15.6 months; *p* < 0.001). **(c)** Exemplary photograph of serous ovarian cancer without ACTBL2-positive tumor-infiltrating leukocytes. **(d–h)** Detection of ACTBL2-positive TILs by immunohistochemistry. Representative photographs of all considered histological subtypes in the given study. Throughout all specimens, the cytoplasmic ACTBL2 expression in leukocytes was remarkably higher than the ACTBL2 expression in tumor cells of **(d)** high-grade serous, **(e)** low-grade serous, **(f)** clear cell, **(g)** mucinous and **(h)** endometrioid ovarian carcinoma (x25 magnification, scale bar = 100µm).

Intending to investigate the distribution of ACTBL2-positive tumor-infiltrating leukocytes, immunohistochemical staining was analyzed in a cohort of 156 ovarian cancer patients. Tissue of 128 patients (82% of all evaluable cases) could be assessed regarding the specific presence of ACTBL2-expressing TILs, being binarily divided into two groups. In 99 out of 128 cases, an infiltration by ACTBL2-positive leukocytes was detected (77%), whereas in 29 cases (23%) such intra-tumoral leukocyte spreading could not be shown (Fig. 1c). Representative photographs of ACTBL2-expressing TILs in all considered histological subtypes are depicted in Fig. 1d–h, demonstrating that the staining intensity and thus cytoplasmic ACTBL2 expression in leukocytes was remarkably higher than the ACTBL2 level in tumor cells throughout all specimens.

Consecutively performed correlation analyses with clinicopathological data revealed a significant negative correlation between patients’ age and the occurrence of ACTBL2-positive TILs (Table S3; *Cc* = − 0.226, *p* = 0.009). In addition, an infiltration by ACTBL2-expressing leukocytes was significantly associated with low grading of serous carcinoma (Table S3; *Cc* = 0.200, *p* = 0.025).

Aiming to further delineate the prognostic significance of ACTBL2-expressing leukocytes in EOC, a univariate analysis regarding the overall survival (OS) was executed. The patients’ median age in the present cohort (n = 156) was 58.7 (standard deviation (SD) = 31.4) years with a range from 20.7–88.0 years, while their median OS amounted 33.8 (SD = 57.8) months. A tumor infiltration by ACTBL2-positive leukocytes was found to be significantly associated with improved overall survival of EOC patients (Fig. 1a; 61.2 (n = 99) vs. 34.4 (n = 29) months; *p* = 0.006).

Considering the previously described significant correlation with low-grade serous histology, the impact of ACTBL2-expressing leukocytes on patients’ OS in this distinct subgroup (n = 24) was further evaluated. The median age of the corresponding patients was 50.0 (SD = 13.4) years with a median survival time of 105.4 (SD = 63.6) months. Emphasizing the outlined results concerning the overall collective, a highly significant prognostic benefit of patients with detectable ACTBL2-positive TILs and low-grade serous carcinoma (LGSC) was revealed (Fig. 1b; median not reached (n = 17) vs. 15.6 months (n = 4); *p* < 0.001).

### The presence of ACTBL2-expressing TILs is an independent prognostic factor for overall survival

For the detection of independent prognostic factors for overall survival in the analyzed cohort, a multivariate Cox regression analysis was executed (Table 2). Histological grading (Hazard Ratio (HR) = 1.841, *p* = 0.001), as well as FIGO stage (HR = 2.099, *p* < 0.001), were confirmed as statistically independent factors in the overall collective. Moreover, the presence of ACTBL2-positive TILs in EOC patients’ tissue, regardless of the histological subtype, was found to be a novel and independent prognostic factor for overall survival with a Hazard Ratio of 0.556 (*p* = 0.038). Since an infiltration by ACTBL2-expressing immune cells was shown to play a significant prognostic role especially in patients with LGSC, a separate Cox regression was calculated for this specific subgroup. In line with the obtained results concerning the total cohort, an infiltration by ACTBL2-positive leukocytes was identified as a statistically independent prognostic marker for the overall survival of patients with low-grade serous ovarian cancer (HR = 0.058, *p* = 0.018).

**Table 2:** Multivariate analysis. Multivariate Cox regression analysis regarding the overall collective (n = 156) and the subgroup of patients with low-grade serous carcinoma (LGSC; n = 24) and their clinicopathological features as considered in the present study. As the distinct cases of patients with LGSC have been pre-selected before executing the calculation, data on histology and grading are consequently not available. Significant independent prognostic factors for overall survival in this cohort are indicated with asterisks (*p < 0.05; **p < 0.001).

Covariate	Overall collective			Low-grade serous carcinoma		
	Hazard Ratio	95% CI	*p* value	Hazard Ratio	95% CI	*p* value
Patients’ age (≤ 60 vs. > 60)	1.301	0.804–2.106	0.284	1.909	0.428–8.521	0.397
Histology	0.960	0.710–1.297	0.789	–	–	–
Grading	1.841	1.275–2.660	0.001*	–	–	–
FIGO stage	2.099	1.402–3.145	< 0.001**	1.600	0.557–4.597	0.383
ACTBL2-positive TILS	0.556	0.319–0.969	0.038*	0.058	0.005–0.618	0.018*

### ACTBL2-positive TILs in EOC are mainly composed of CD44-positive cytotoxic T-cells and macrophages

**Figure 2:**
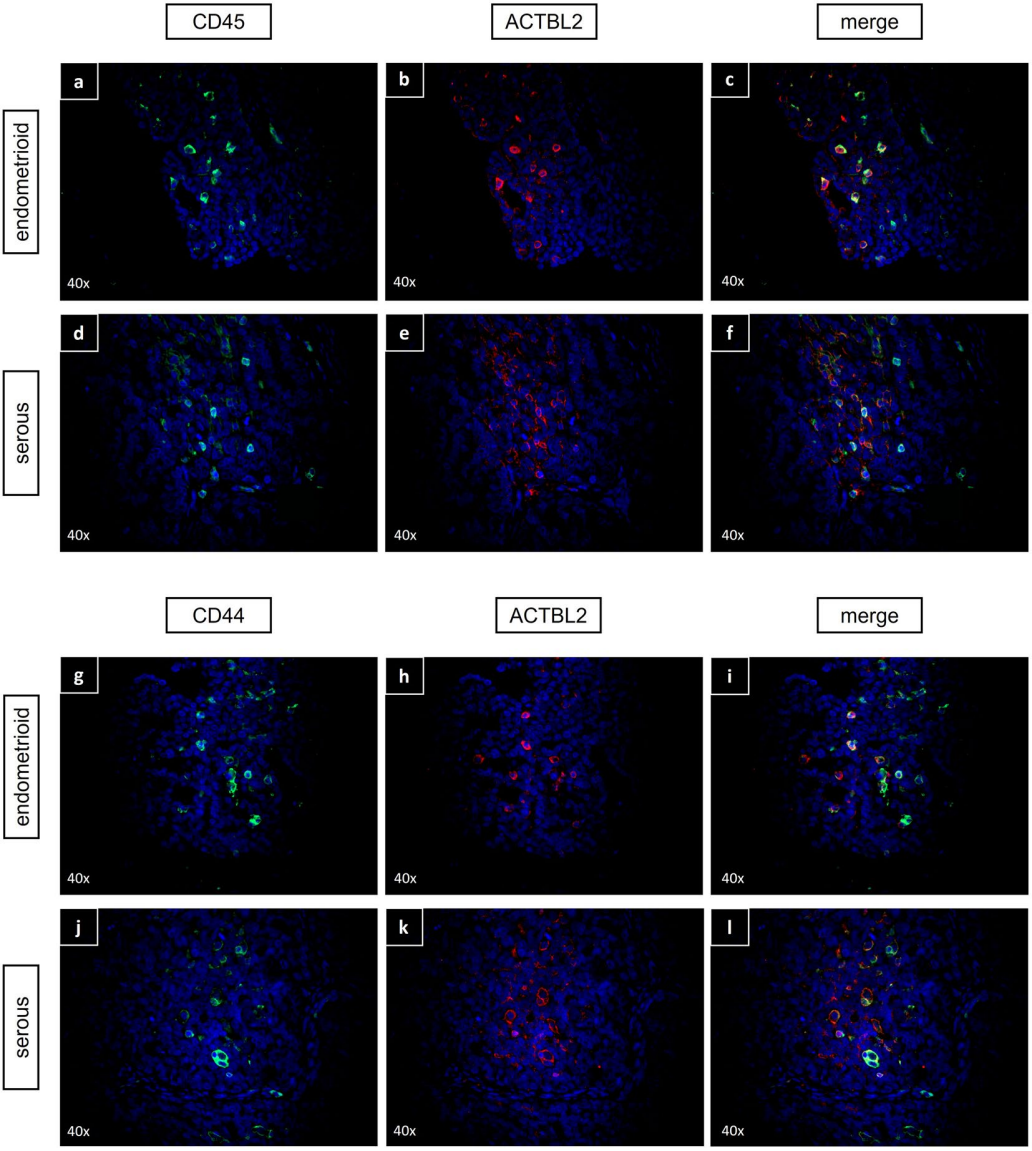
Immunofluorescence double-staining with anti-ACTBL2, anti-CD45 and anti-CD44 antibodies. (**a–f**) Representative staining results of EOC tissue of endometrioid (**a–c**) and serous (**d–f**) histology, showing a high ACTBL2 expression in CD45-positive tumor-infiltrating leukocytes. **(g–l)** Exemplary photographs of endometrioid (**g–i**) and serous (**j–l**) carcinoma, proving a co-expression of ACTBL2 and membrane-bound CD44 as a marker for activated TILs (×40 magnification).

**Figure 3:**
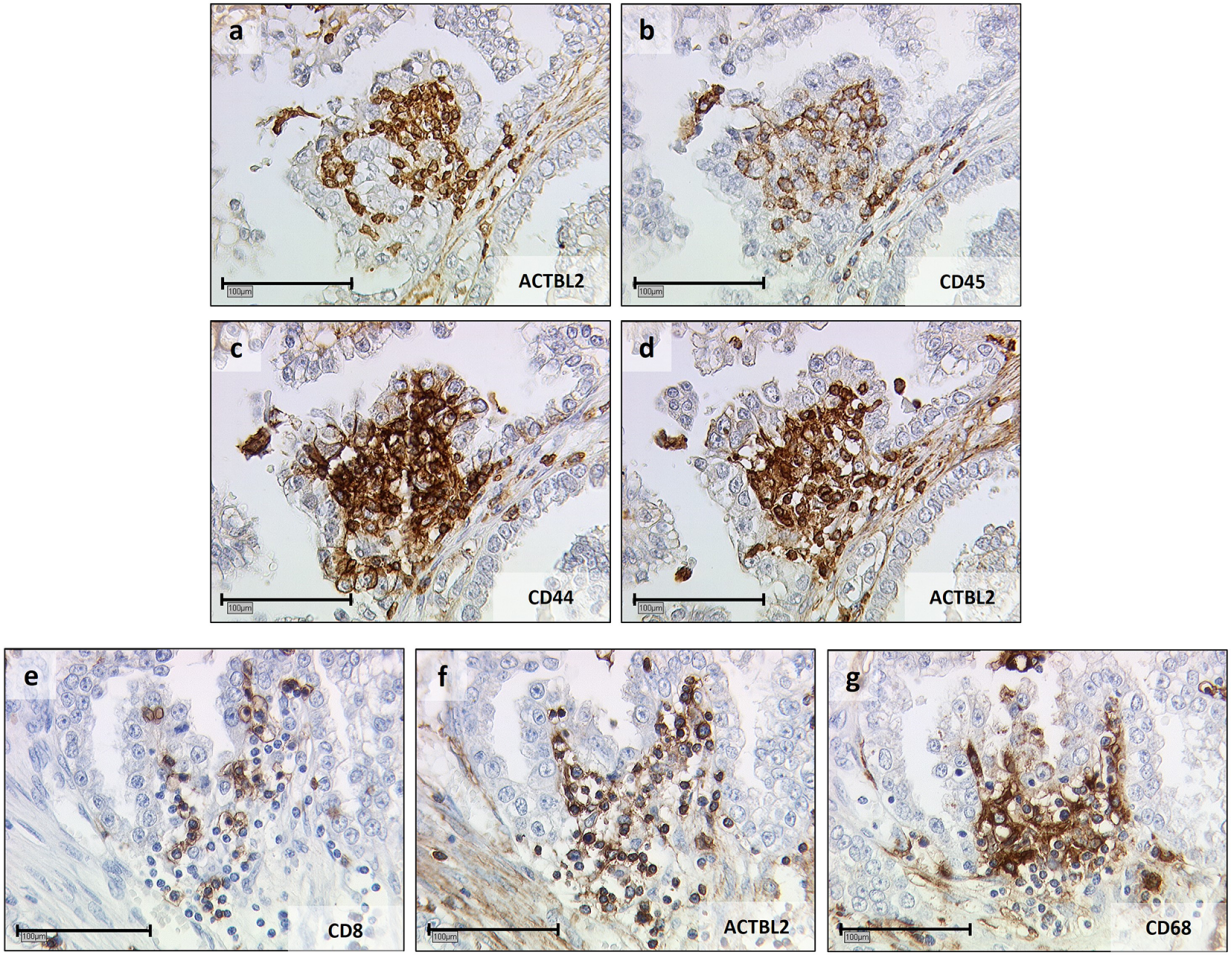
Serial staining of clear cell carcinoma tissue for leukocyte subtyping. **(a–d)** Exemplary photographs of consecutive clear cell carcinoma tissue slices after immunohistochemical staining of ACTBL2 (**a, d**), CD45 (**b**) and CD44 (**c**), identifying ACTBL2-overexpressing cells as tumor-infiltrating CD44-positive leukocytes. **(e–g)** Representative pictures of another clear cell carcinoma series, revealing CD8-positive cytotoxic T-cells (**e**) and CD68-positive macrophages (**g**) as the predominant cellular subsets of ACTBL2-positive TILs (**f**) (×25 magnification, scale bar = 100 µm).

**Figure 4:**
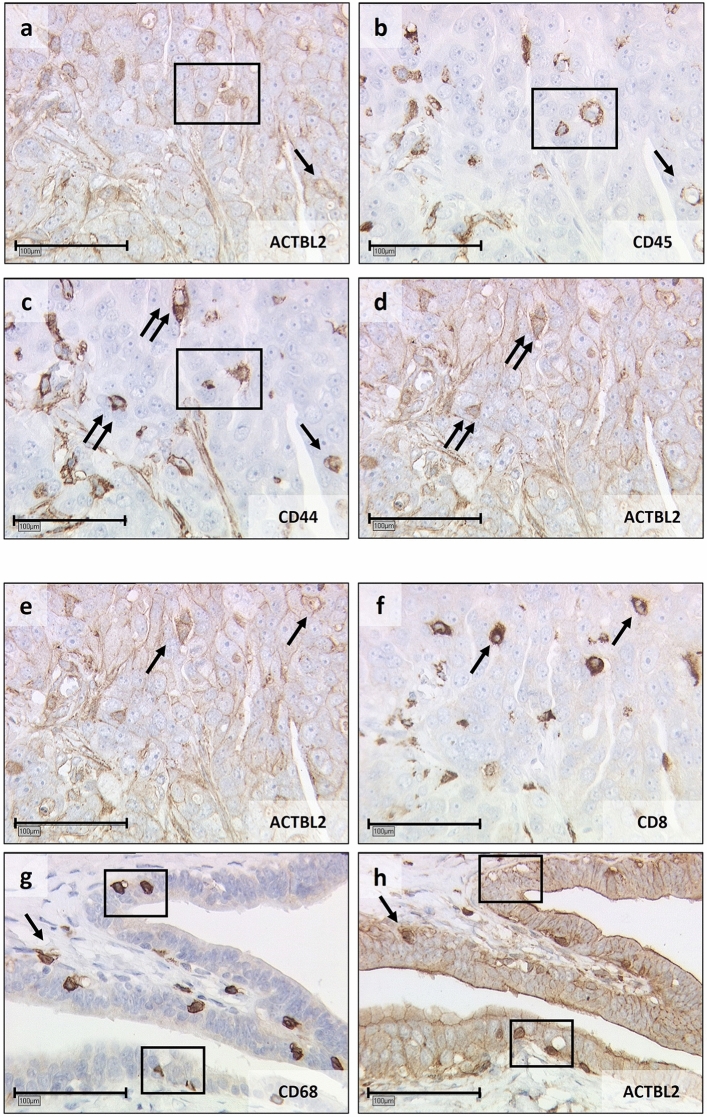
Serial staining of serous fallopian tube cancer for leukocyte subtyping.** (a–d)** Representative photographs of an immunohistochemical staining series of serous fallopian tube cancer, hinting at the co-expression of CD45 (**b**) and CD44 (**c**) by ACTBL2-positive (**a, d**) immune cells. **(e–h)** Exemplary pictures of two further series, identifying ACTBL2-overexpressing leukocytes (e and h) as CD8-positive (**f**) and CD68-expressing (**g**) immune cells. Identical cells between the pictures were marked by rectangles and arrows (×25 magnification, scale bar = 100 µm).

Aiming at characterizing the subset of ACTBL2-expressing TILs in epithelial ovarian cancer with special regard to putative immune-mediated antitumoral effects, immunofluorescence double-staining was performed. In the first step, exemplary tissue slides of each histological subtype with previously detected leukocyte infiltration in the corresponding TMA were double-stained with antibodies against ACTBL2 and CD45 as a common leukocyte antigen to demonstrate the high protein expression of ACTBL2 in TILs in general (Fig. 2a–f). Assuming that ACTBL2 plays a significant role in cellular motility and migration of activated leukocytes, CD44 as a commonly known adhesion molecule and regulator of intra-tumoral leukocyte movement^39,40^ was chosen for subsequent analyses. As depicted in Fig. 2g–l, ACTBL2-positive cells showed a clear co-expression of CD44. For an even more precise cellular characterization, serial tissue slides of the according specimens were produced, thus enabling a consecutive immunohistochemical staining series with up to four antibodies and an exact identification of singular cells in each histological subtype. As exemplarily shown in Fig. 3a–d for clear cell and Fig. 4a–d for serous carcinoma, tumor-infiltrating leukocytes showed a high intracellular level of ACTBL2 as well as a strong expression of membrane-bound CD44. Intending to define the exact cellular composition of ACTBL2-expressing leukocytes in ovarian cancer, various common markers for the most frequent immune cell subtypes have been investigated—CD4 (T-helper cells), FOXP3 (regulatory T-cells) and CD56 for natural killer cells. None of the mentioned markers could be detected in ACTBL2-positive TILs (data not shown). Instead, complementary serial staining identified CD8-expressing cytotoxic T-cells and CD68-positive macrophages as the predominant cellular subtypes (Figs. 3e–g, 4e–h). Further exemplary photographs and results of immunofluorescence double-staining as well as immunohistochemical serial staining of all remaining histological EOC subtypes are shown in Figures S3–S6.

## Discussion

Recent studies provided growing evidence on the impact of Actin beta-like 2 on cellular motility by elucidating its molecular function in the course of lamellipodia formation. Comprehensive analyses of human melanoma cells revealed a specific interaction between polymerized ACTBL2 and the multifunctional actin-binding protein gelsolin in the edge of lamellipodia as protrusions conducting cellular migration^24,41^. Moreover, Malek et al. proved a significant change in the actin cytoskeleton structure upon knockout of *ACTBL2* resulting in impaired cellular invasion abilities and altered invadopodia and focal adhesion formation^23^. Despite the given findings, the extent to which ACTBL2 is expressed in tumor-infiltrating leukocytes as cells with high migratory potential and activity remains to date unknown.

The present study is the first one to ever investigate the expression of ACTBL2 in TILs with special regard to their prognostic significance on the overall survival of ovarian cancer patients. By examining the presence of ACTBL2-positive TILs in 156 EOC specimens via immunohistochemistry we could demonstrate that the expression of ACTBL2 in leukocytes was remarkably higher than its level in the according tumor cells. Subsequent survival analyses confirmed a significant and statistically independent prognostic benefit for patients with detectable ACTBL2-expressing TILs. Assuming that the composition of the given leukocyte subset is crucial for the favorable prognosis, further comprehensive staining series were executed to precisely define the distinct cellular subtypes in terms of immune-mediated antitumoral effects. Since effective leukocyte homing is indispensable for a successful tumor invasion, we consequently examined the co-expression of ACTBL2 and CD44 in TILs, presuming a significant interplay between cellular adhesion provided by CD44 and a consecutive promigratory rearrangement of the actin cytoskeleton.

Mrass et al. showed that CD44, a surface glycoprotein and adhesion receptor for extracellular matrix (ECM) proteins and glycosaminoglycans, is localized in small cellular cell protrusions at the rear end of crawling T-cells^39^. Whereas its extracellular domain is essential for a close interaction with ECM fibers by promoting cellular attachment, the intracellular domain was shown to contain several binding sites for signaling molecules, providing a linkage between the actin cytoskeleton and membrane proteins and thus regulating cell migration^39,42,43^. Consequently, CD44-deficient cytotoxic T cells showed a significantly reduced migratory potential, indicating its substantial impact on cellular polarity and intra-tumoral navigation^39^. Focusing on cellular structures enhancing motility, CD44s as a specific splice isoform was identified as an integral element in invadopodia of tumor cells^44^. Invadopodia are dot-shaped and actin-rich protrusions with the ability to degrade the ECM, hence enabling the invasion of cancer cells^45^. Zhao et al. stated a significantly suppressed invadopodia activity and diminished invasiveness upon shRNA-mediated depletion of CD44s^44^. Podosomes, similar protrusive structures with an actin-rich core, are mainly located at the ventral site of e.g. vascular smooth muscle cells and antigen-presenting cells (APC) like dendritic cells (DC) and macrophages^45^. Culturing of the respective cells in collagen resulted in the formation of filamentous actin-rich protrusions containing accumulated podosome-associated proteins such as ß1-integrin and gelsolin as well as CD44, which was significantly associated with the proteolytic activity of human macrophages^45,46^. Consistent with the given evidence of the significant molecular impact of CD44 on the rearrangement of the actin cytoskeleton as conveyed by distinct cellular protrusions, we could prove that ACTBL2-positive TILs show a high co-expression of CD44, potentially hinting at a direct interaction in the course of leukocyte migration and activation.

Aiming at a particular identification of the predominant cell types and the accordingly conveyed tumor-modulating effects, additional staining analyses of the present EOC patient cohort defined the subset of ACTBL2-positive TILs as CD44-expressing cytotoxic T-cells (CD8 +) and macrophages (CD68 +). Besides being involved in the homing of leukocytes and antigen-presenting cells to sites of inflammation, CD44 was shown to directly mediate the lytic and anti-tumoral activity of cytotoxic T-cells by promoting specific transmembrane signals leading to granule exocytosis^47,48^. Further, Hegde et al. demonstrated that CD44 clustered at the contact between T-cells and mature DCs in the course of T-cell activation, mediating the formation of an immunological synapse^48^*.* The direct interaction of both cells resulted in an accumulation of F-actin within the T-cell at the specific binding point with a consecutive re-arrangement of the actin cytoskeleton, increasing the contact between the T-cell receptor and MHC-receptors on DCs^49^. Zhang et al. showed that the presence of intra-tumoral T-cells was an independent prognostic factor for overall and progression-free survival in ovarian cancer^50^. More specifically, intraepithelial CD8 + TILs and a high cytotoxic T-cell/regulatory T-cell (Treg) ratio were associated with a prognostic benefit for EOC patients^51^. Apart from the aforementioned mechanisms, tumor-associated macrophages (TAM) play a crucial role in shaping the distinct tumor environment by producing different cytokines and executing contrary functions depending on their polarization status^52,53^. Ovarian cancer patients with an increased M1/M2 TAM ratio in favor of tumor-suppressive and pro-inflammatory M1 macrophages showed a significantly improved overall survival^54,55^. However, ovarian cancer cells were shown to directly influence the polarization status of TAMs by changing it into the M2-like phenotype, which consequently is the predominant one in EOC and suppresses an effective cytotoxic T-cell response by fostering Treg recruitment^52,56,57^. In contrast, Paclitaxel was shown to reprogram M2-polarized macrophages to the M1-like phenotype as a part of its therapeutic effect^58^.

Taken together, our results suggest that the favorable prognostic effect of ACTBL2-expressing TILs in ovarian cancer is attributed to the presence and close interaction of activated cytotoxic T-cells and macrophages, probably in their function as antigen-presenting and T-cell-attracting cells. Additional analyses are required for a further distinction of the macrophage polarization status and the presence of dendritic cells in order to completely elucidate the definite cellular composition and interplay. As ACTBL2 and CD44, executing a key role in focal adhesion and T-cell activation, were both shown to closely interact with gelsolin in the course of cytoskeleton alteration, ACTBL2 might represent a novel marker for migrating and activated immune cells. Since sufficient tumor infiltration by T-cells is mandatory for a successful immune checkpoint blockade^59^, the identification of ACTBL2-positive CD44 + /CD8 + TILs in EOC tissue might be a means to determine potential patient subgroups, being particularly prone to respond to immune therapy.

Interestingly, correlation analyses showed a particular survival benefit for patients with LGSC and ACTBL2-positive TILs despite a low number of cases. Kurman et al. postulated a dualistic model, claiming that LGSC is a separate tumor entity due to specific histological characteristics and substantial molecular differences^7,30,60^. Patients with LGSC are characterized by a younger age at diagnosis and a better overall survival despite showing a relative resistance against platinum-taxane-based therapy due to its lower mitotic rate, warranting alternative and subtype-specific treatment strategies^61,62^. Since the majority of LGSC shows a positive estrogen receptor expression, hormone-based maintenance therapy revealed a promising effect on median progression-free survival compared to patients only undergoing clinical observation^63^. Other therapeutic options include the use of CDK4/6 inhibitors^61^ and MEK inhibitor Trametinib for recurrent disease since the MAP-kinase pathway was shown to be a crucial part of LGSC pathogenesis^64,65^. To date, comprehensive analyses regarding the tumor microenvironment of LGSC considering the use and efficacy of immune checkpoint inhibition are missing, presumably because of its comparably rare occurrence. Milne et al. stated that the presence of TILs varied significantly between different subtypes of EOC, with HGSC showing the highest frequency of intra-tumoral CD45 + cells, but without taking LGSC into consideration^66^. Lacking TP53 or BRCA1/2 mutation and genomic stability further contribute to a reduced neo-antigen presentation in LGSC^67^. Additional detailed examinations of a larger number of cases are required to provide further knowledge on the unique composition of the tumor environment of LGSC and to elucidate the potential of immune therapeutical approaches.

Concluding, our study provides for the first time significant evidence of the favorable prognostic impact of ACTBL2-expressing TILs in epithelial ovarian cancer with regard to different histological subtypes. Actin beta-like 2 as a new and additional actin isoform may execute crucial functions in terms of leukocyte motility and activation by interacting with CD44 during cytoskeleton reorganization. Complementary studies are required to further analyze the role of ACTBL2 as a putative marker for activated migrating leukocytes on a molecular basis and to characterize the immunological impact of ACTBL2-positive TILs on ovarian carcinogenesis.

### Supplementary Information

Supplementary Information.

## Data Availability

The original contributions presented in the study are included in the article/the corresponding Supplementary Material. Further inquiries can be directed to the corresponding author.

## Abbreviations

ACTBL2: Actin beta-like 2
APC: Antigen-presenting cell
BRCA1/2: BReast CAncer 1/2
CI: Confidence interval
Cc: Correlation coefficient
CDK 4/6: Cycline-dependent kinase 4/6
cDNA: Complementary desoxy-ribonucleic acid
DAB: 3,3′Diaminobenzidine
ECM: Extracellular matrix
EOC: Epithelial ovarian cancer
F-actin: Filamentous actin
FBS: Fetal bovine serum
FFPE: Formalin-fixed and paraffin-embedded
FIGO: International Federation of Gynecology and Obstetrics
HGSC: High-grade serous carcinoma
HR: Hazard Ratio
HRD: Homologous recombination deficiency
ICC: Immunocytochemistry
IF: Immunofluorescence
IHC: Immunohistochemistry
LGSC: Low-grade serous carcinoma
LMU: Ludwig-Maximilians-University
MAP-kinase: Mitogen-activated protein kinase
MCR: Munich Cancer Registry
mRNA: Messenger ribonucleic acid
NFAT5: Nuclear factor of activated T-cells 5
OS: Overall survival
PARPi: Poly-ADP-ribose-polymerase inhibitor
PBS: Phosphate-buffered saline
qPCR: Quantitative polymerase chain reaction
RNA: Ribonucleic acid
ROC: Receiver operating characteristic
RT: Room temperature
SD: Standard deviation
siRNA: Small interfering ribonucleic acid
TAM: Tumor-associated macrophage
TILs: Tumor-infiltrating leukocytes
TMA: Tissue-Microarray
TMB: Tumor mutational burden
VSMC: Vascular smooth muscle cell
WHO: World Health Organization
