# Supplementary material for: Characterization and prognostic impact of ACTBL2-positive tumor-infiltrating leukocytes in epithelial ovarian cancer
Source: Sci Rep. 2023 Dec 18;13:22620. doi: 10.1038/s41598-023-49286-9 (PMC10730610; doi:10.1038/s41598-023-49286-9)
Supplement: Supplementary file 1 — Supplementary Information. [file 41598_2023_49286_MOESM1_ESM.docx]

**Supplementary File to:** **Characterization and prognostic impact of ACTBL2-positive tumor-infiltrating leukocytes in epithelial ovarian cancer**

Topalov NE^1,*^, Mayr D^2^, Kuhn C^3^, Leutbecher A^4,5^, Scherer C^6^, Kraus FBT^1^, Tauber CV^1^, Beyer S^1^, Meister S^1^, Hester A^1^, Kolben T^1^, Burges A^1^, Mahner S^1^, Trillsch F^1^, Kessler M^1^, Jeschke U^1,3^ and Czogalla B^1^

| Antibody | Species | Dilution | Incubation | Manufacturer | Catalog number |
| --- | --- | --- | --- | --- | --- |
| Anti-ACTBL2 | rabbit IgG  polyclonal | 1:800 (IHC, IF)  1:400 (ICC) | 16h at 4°C | Abcam, Cambridge, UK | ab100869 |
| Anti-CD45 | mouse IgG1 kappa monoclonal | 1:200 | 16h at 4°C | DAKO, Glostrup, Denmark | M0701 |
| Anti-CD44 | mouse IgG2 kappa monoclonal | 1:5000 (IHC)  1:2000 (IF) | 30 minutes at RT | Abcam, Cambridge, UK | ab213072 |
| Anti-CD8 | mouse IgG monoclonal | 1:200 | 16h at 4°C | Zytomed, Berlin, Germany | Mob117-01 |
| Anti-CD68 | mouse IgG1 monoclonal | 1:2000 | 16h at 4°C | Sigma-Aldrich, St.Louis, MO, USA | AMAb90874 |
| Antibody diluent for IHC and ICC: PBS  Antibody diluent for IF: DAKO Antibody Diluent (REF S3022); DAKO, Glostrup, Denmark | | | | | |

**Table S1**| Primary antibodies and corresponding dilutions used for immunohistochemistry (IHC), immunocytochemistry (ICC) and immunofluorescence (IF) staining.

| Antibody | Dilution | Incubation | Manufacturer |
| --- | --- | --- | --- |
| Cy3-conjugated goat-anti-rabbit IgG | 1:500 | 30 minutes at RT | Dianova, Hamburg, Germany |
| Cy2-conjugated goat-anti-mouse IgG | 1:100 | 30 minutes at RT | Dianova, Hamburg, Germany |

**Table S2**| Secondary antibodies and corresponding dilutions used for immunofluorescence staining.


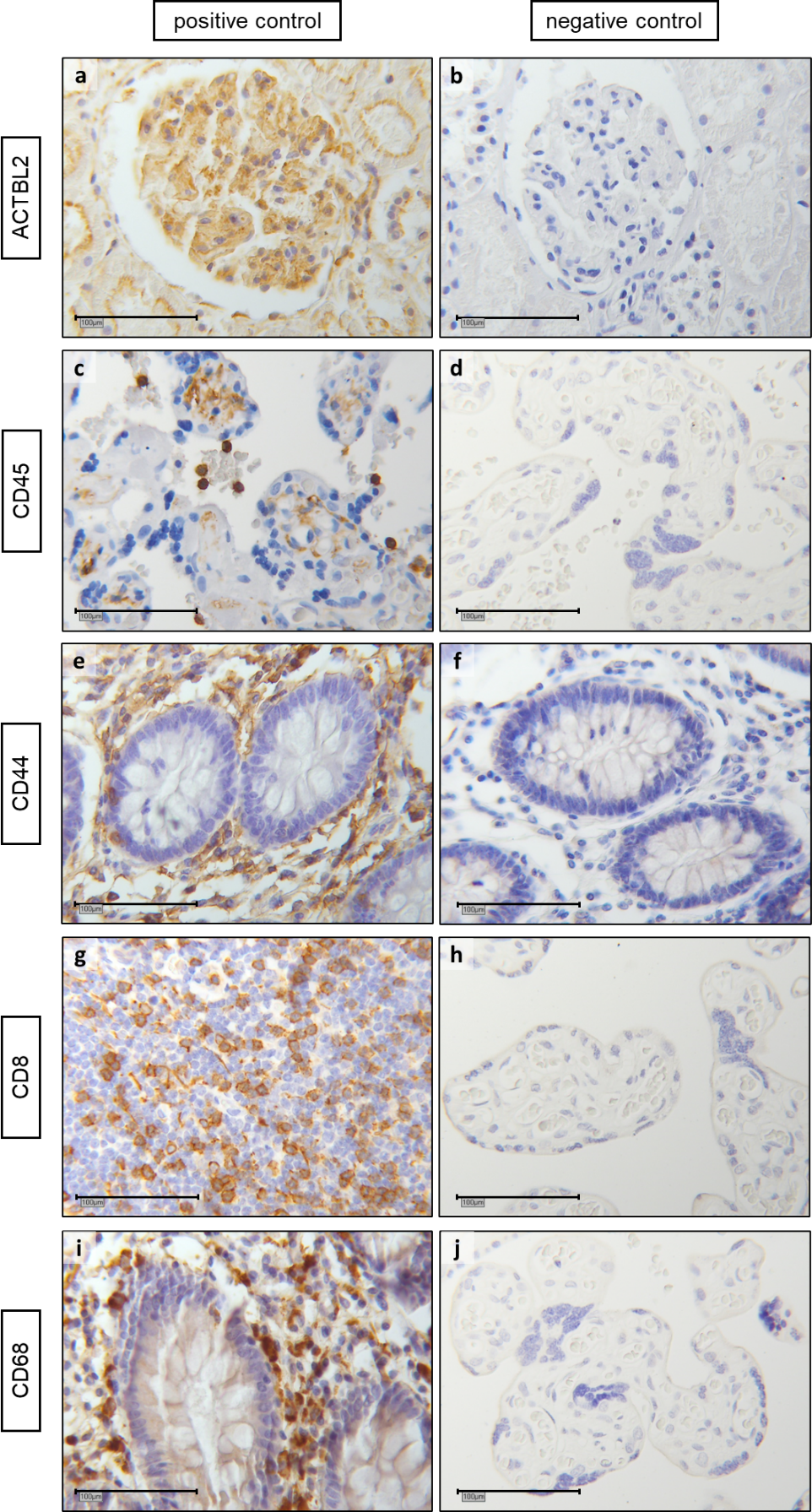


**Figure S1| Positive and negative system controls for immunohistochemistry staining.**

**(a,b)** Kidney tissue serving as positive (a) and negative (b) system control for ACTBL2 immunostaining. **(c,d)** Placenta tissue serving as positive (c) and negative (d) system control for CD45 immunostaining. **(e,f)** Colon tissue serving as positive (e) and negative (f) system control for CD44 immunostaining. **(g,h)** Tonsil (g) and placenta (h) tissue serving as positive and negative system control for CD8 immunostaining, respectively. **(i,j)** Colon (i) and placenta (j) tissue serving as positive and negative control for CD68 immunostaining, respectively. (25x magnification; scale bar=100µm).


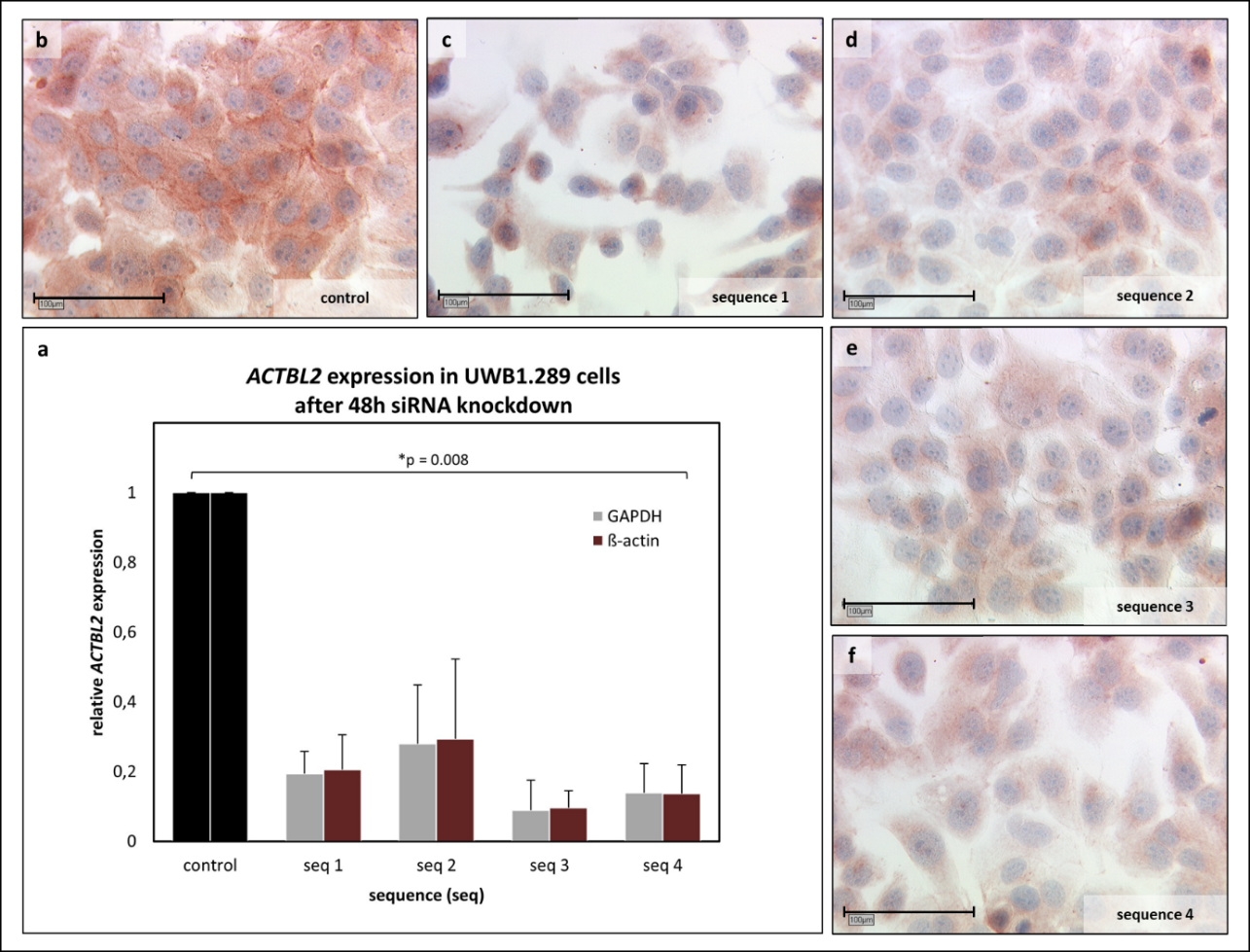

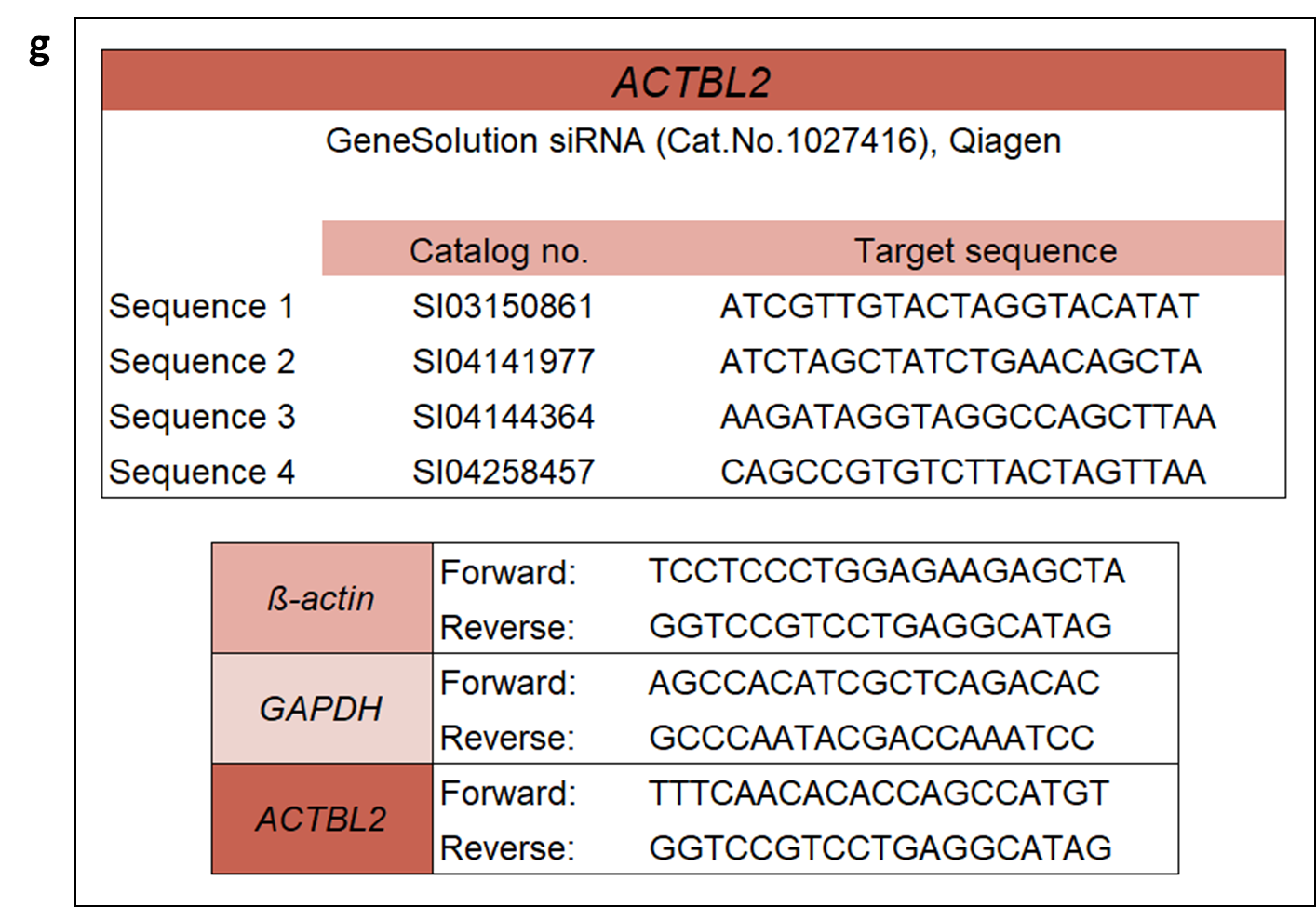


**Figure S2| qPCR and immunocytochemistry results after 48h of siRNA knockdown of *ACTBL2* in UWB1.289 cells, proving the specificity of the used antibody. (a)** qPCR results after siRNA knockdown of *ACTBL2* in UWB1.289 cells, proving a significant decrease in its mRNA expression after 48h of incubation (Wilcoxon test; *p*=0.008). The results with ß-actin and GAPDH as housekeeping genes are shown respectively. **(b-f)** Immunohistochemistry staining of ACTBL2 in UWB1.289 cells after 48h of siRNA knockdown of *ACTBL2,* showing a reduced intracellular protein expression (c-f) compared to the untreated control (b) (25x magnification; scale bar=100µm). **(g)** Catalog numbers and target sequences of the used siRNA for *ACTBL2* knockdown as well as sequences of all qPCR primers (Roche, Basel, Switzerland) used for the determination of the mRNA level of *ß-actin*, *GAPDH* and *ACTBL2*.

|  | **Presence of ACTBL2-positive TILs** | |
| --- | --- | --- |
| **Variables** | **p** | **Correlation coefficient** |
|  |  |  |
| Patients’ age (≤60 vs. >60 years) | **0.009*** | **-0.226** |
| FIGO stage | 0.582 | 0.049 |
| Grading |  |  |
| *serous – low grading* | **0.025*** | **0.200** |
| *serous – high grading* | 0.647 | -0.041 |
| *clear cell, endometrioid and mucinous – G1 to G3* | 0.110 | 0.293 |
|  |  |  |

**Table S3| Correlation analysis regarding the presence of ACTBL2-positive TILs and clinicopathological data of EOC patients.** Spearman’s correlation analysis of tumor-infiltrating ACTBL2-positive leukocytes and clinicopathological characteristics, showing a negative correlation between the occurrence of ACTBL2-expressing TILs in EOC and patients’ age (*Cc*=-0.226, *p*=0.009). Moreover, an infiltration by ACTBL2-positive TILs was significantly associated with low grading of serous ovarian cancer (*Cc*=0.200, *p*=0.025). Significant correlations are indicated with asterisks (*: *p*<0.05). (*p*=two-tailed significance, *Cc*=correlation coefficient).


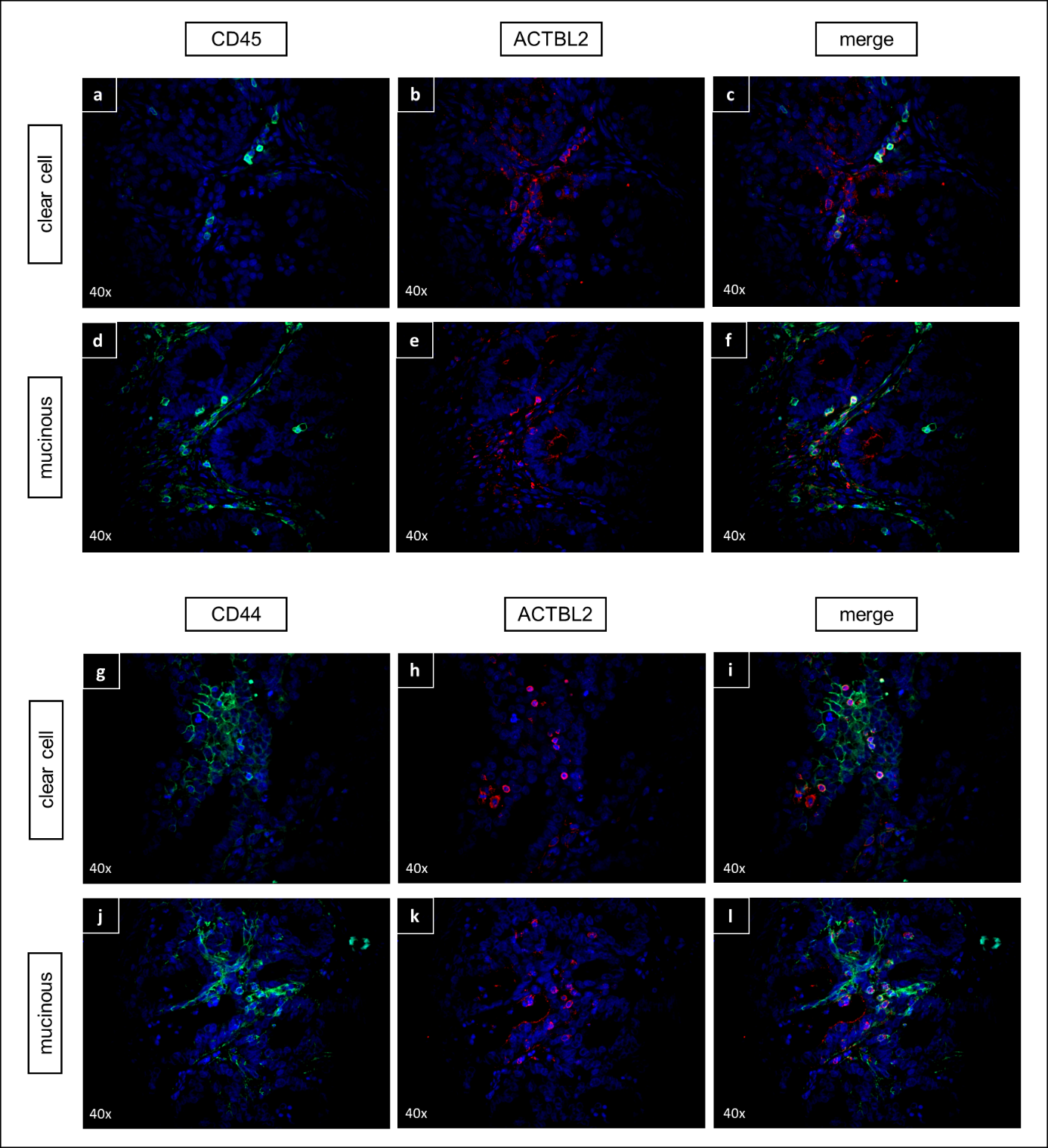


**Figure S3| Immunofluorescence double-staining with anti-ACTBL2, anti-CD45 and anti-CD44 antibodies in clear cell and mucinous ovarian cancer. (a-f)** Representative staining results of EOC tissue of clear cell (a-c) and mucinous (d-f) histology, showing a co-expression of ACTBL2 in CD45-positive tumor-infiltrating leukocytes. **(g-l)** Exemplary photographs of clear cell (g-i) and mucinous (j-l) carcinoma, displaying a co-expression of ACTBL2 and membrane-bound CD44 as a marker for activated TILs (40x magnification).


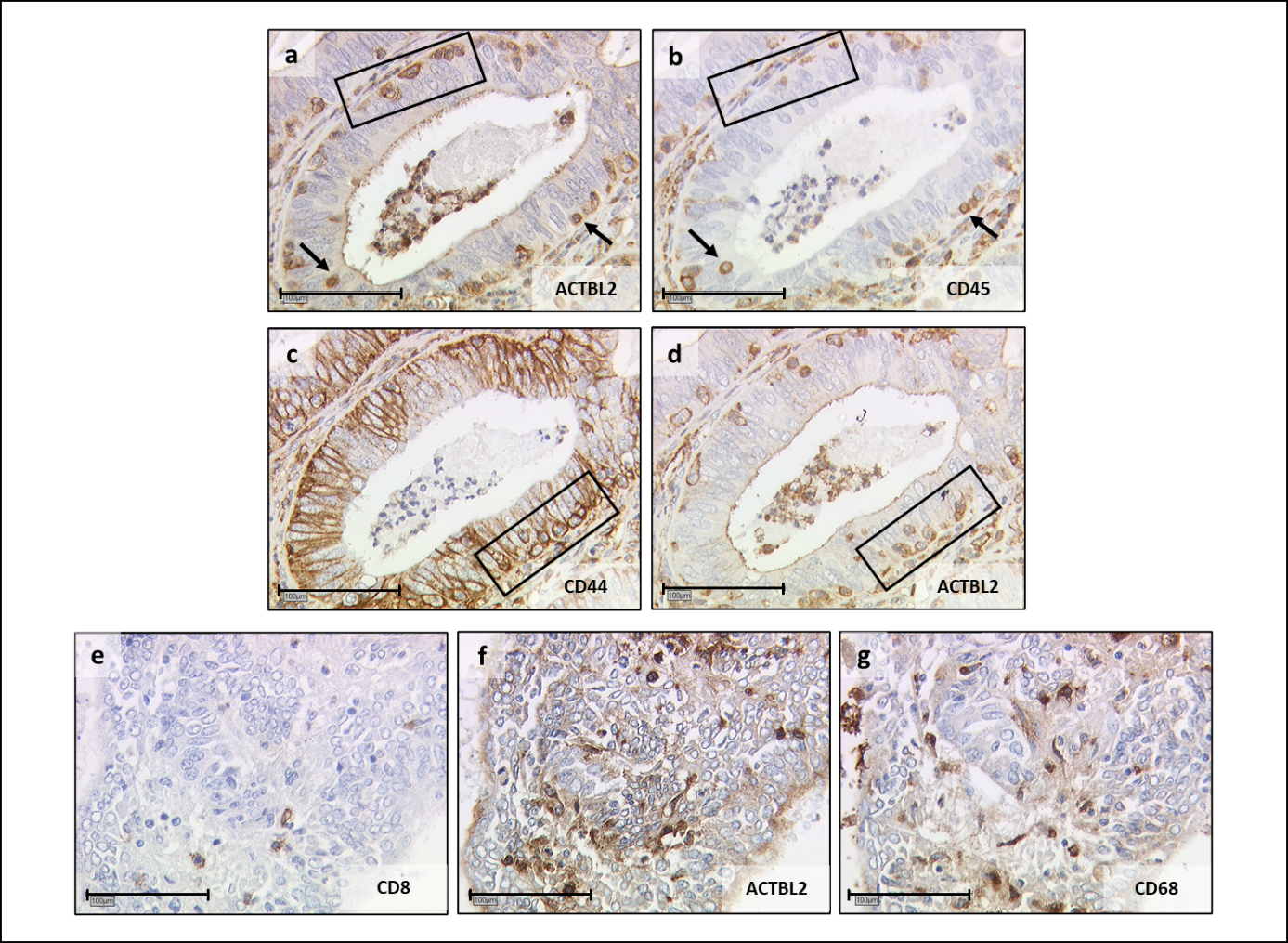


**Figure S4| Serial staining of mucinous carcinoma tissue for leukocyte subtyping. (a-d)** Exemplary photographs of consecutive mucinous carcinoma tissue slices after immunohistochemical staining of ACTBL2 (a and d), CD45 (b) and CD44 (c), identifying ACTBL2-overexpressing cells as tumor-infiltrating CD44-positive leukocytes. **(e-g)** Representative pictures of another mucinous carcinoma series, displaying the CD8 (e) and CD68 (g) expression of ACTBL2-positive TILs (f). Identical cells between the pictures were marked by rectangles and arrows (25x magnification, scale bar=100µm).


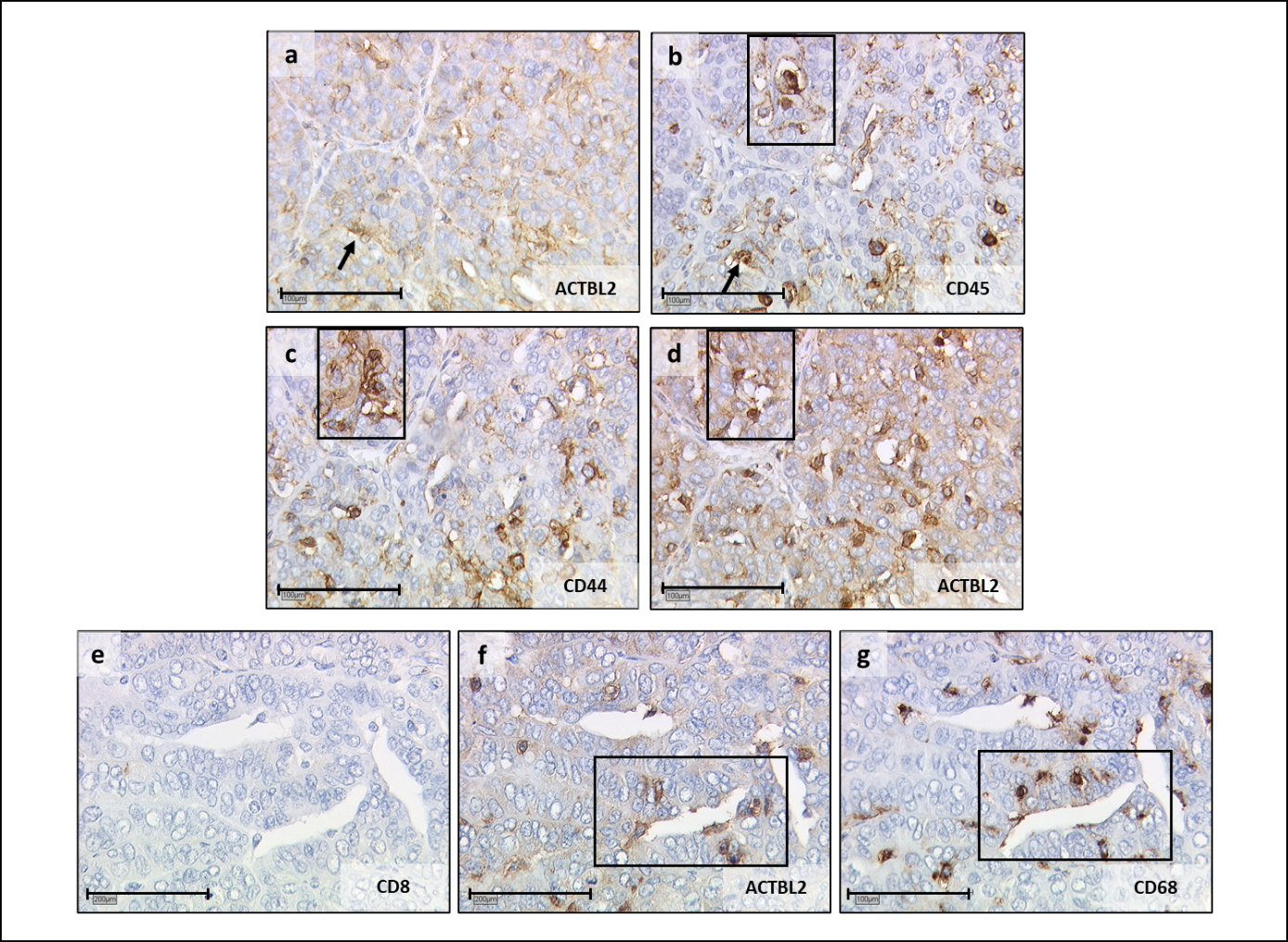


**Figure S5| Serial staining of serous carcinoma tissue for leukocyte subtyping. (a-d)** Exemplary photographs of consecutive serous carcinoma tissue slices after immunohistochemical staining of ACTBL2 (a and d), CD45 (b) and CD44 (c), identifying ACTBL2-overexpressing cells as tumor-infiltrating CD44-positive leukocytes. **(e-g)** Representative pictures of another serous carcinoma series, examining the CD8 (e) and CD68 (g) expression of ACTBL2-positive TILs (f). Identical cells between the pictures were marked by rectangles and arrows (25x magnification, scale bar=100µm).


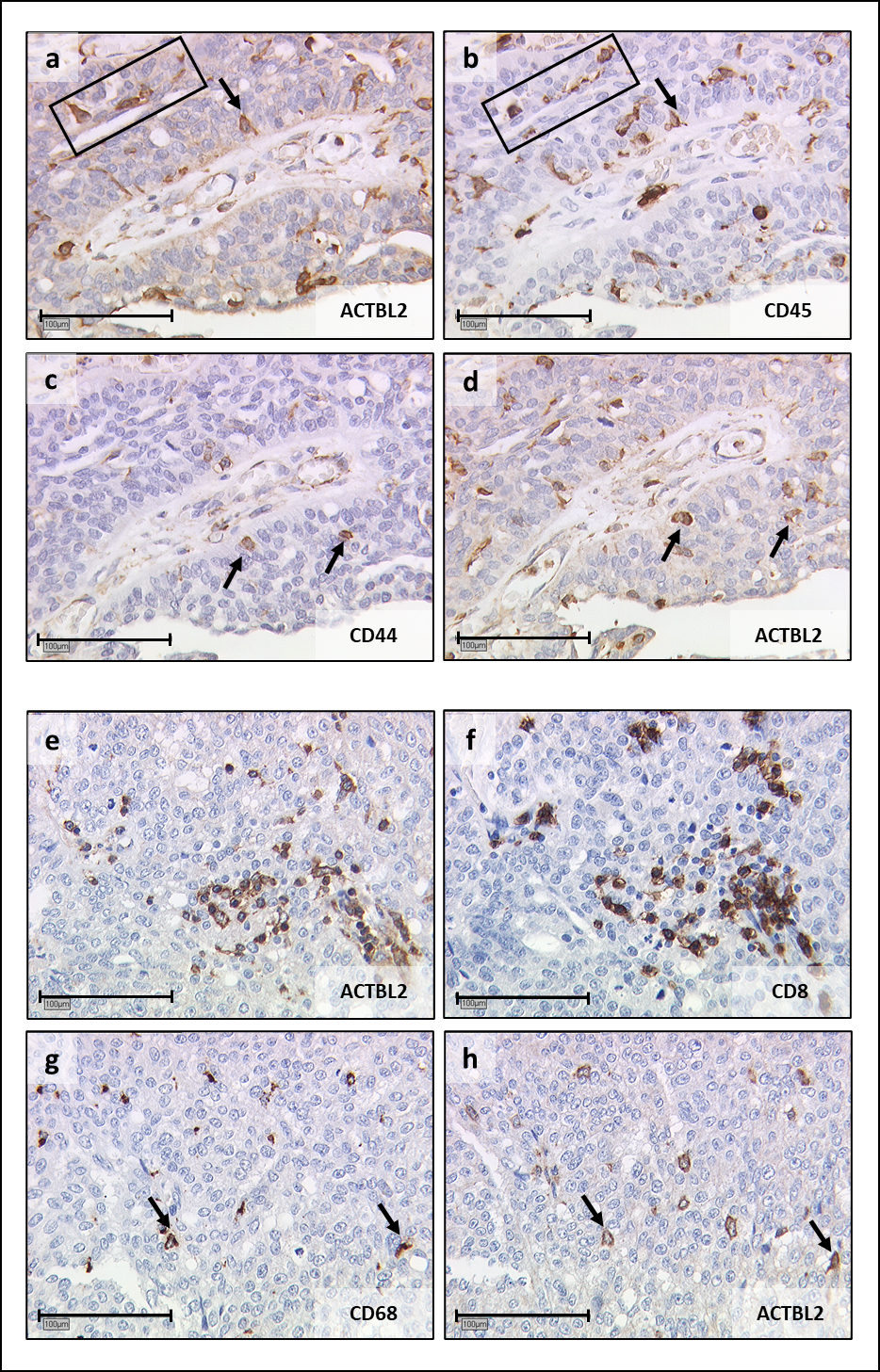


**Figure S6| Serial staining of endometrioid cancer for leukocyte subtyping. (a-d)** Representative photographs of an immunohistochemical staining series of endometrioid cancer, hinting at the co-expression of CD45 (b) and CD44 (c) by ACTBL2-positive (a and d) immune cells. **(e-h)** Exemplary pictures of two further series, identifying ACTBL2-overexpressing leukocytes (e and h) as CD8-positive (f) and CD68-expressing (g) immune cells. Identical cells between the pictures were marked by rectangles and arrows (25x magnification, scale bar=100µm).

Endometrioid


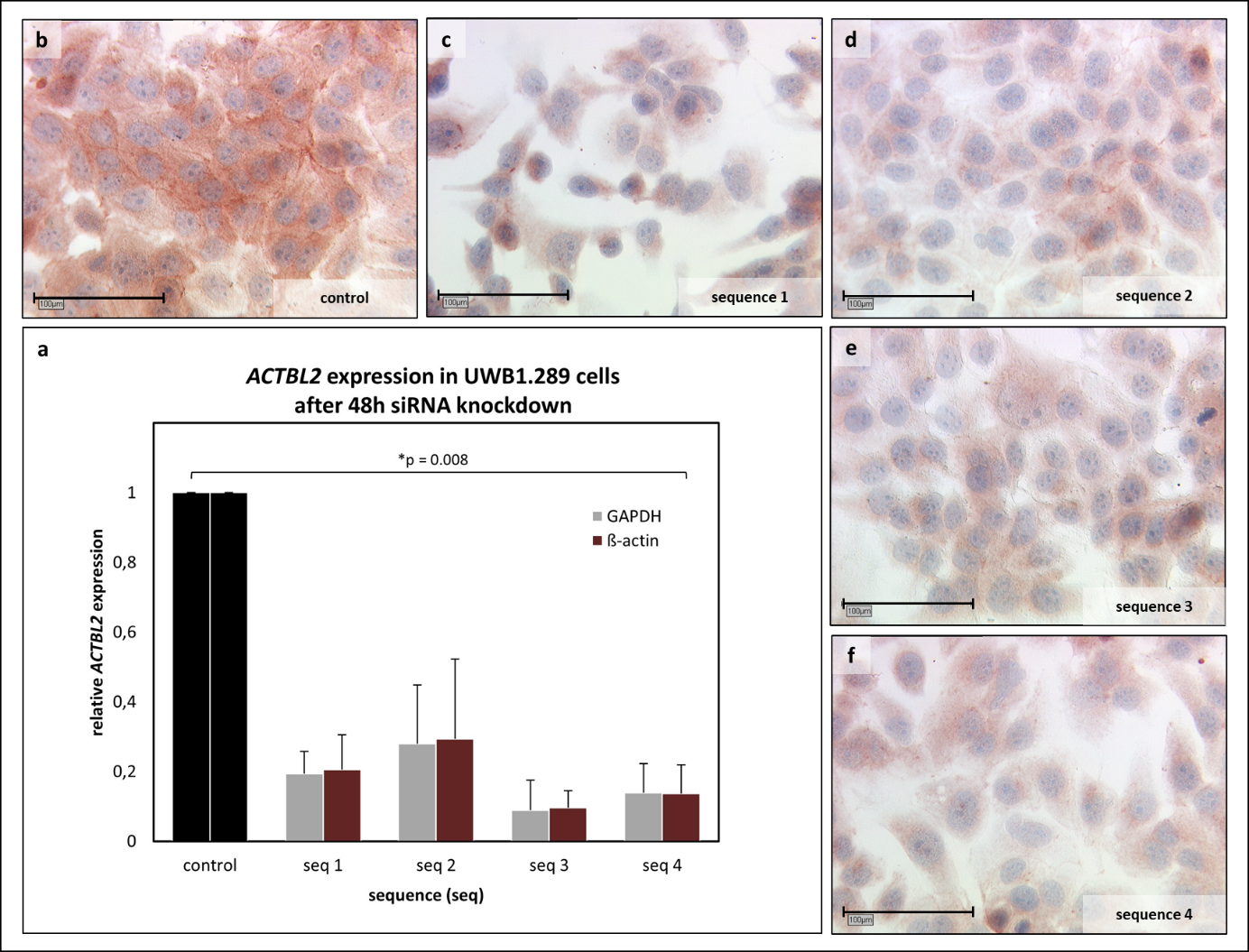


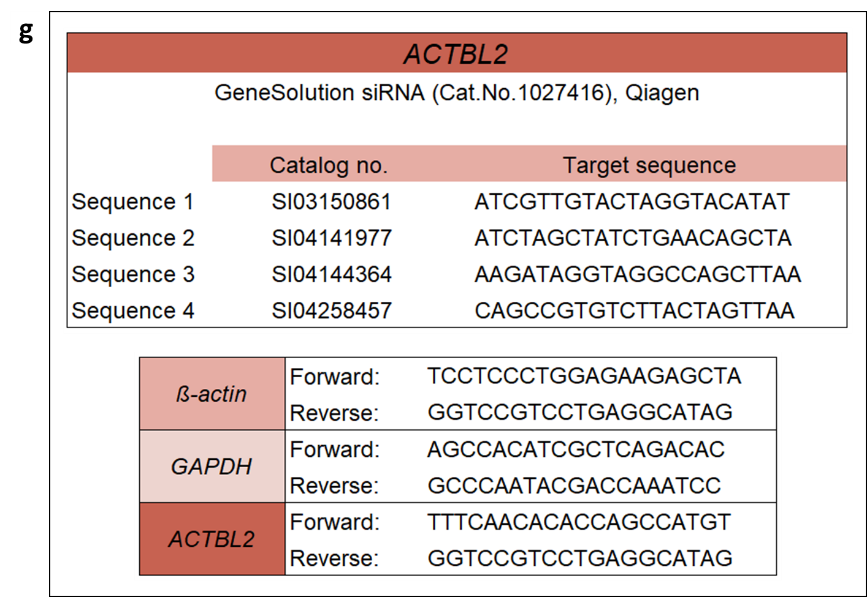


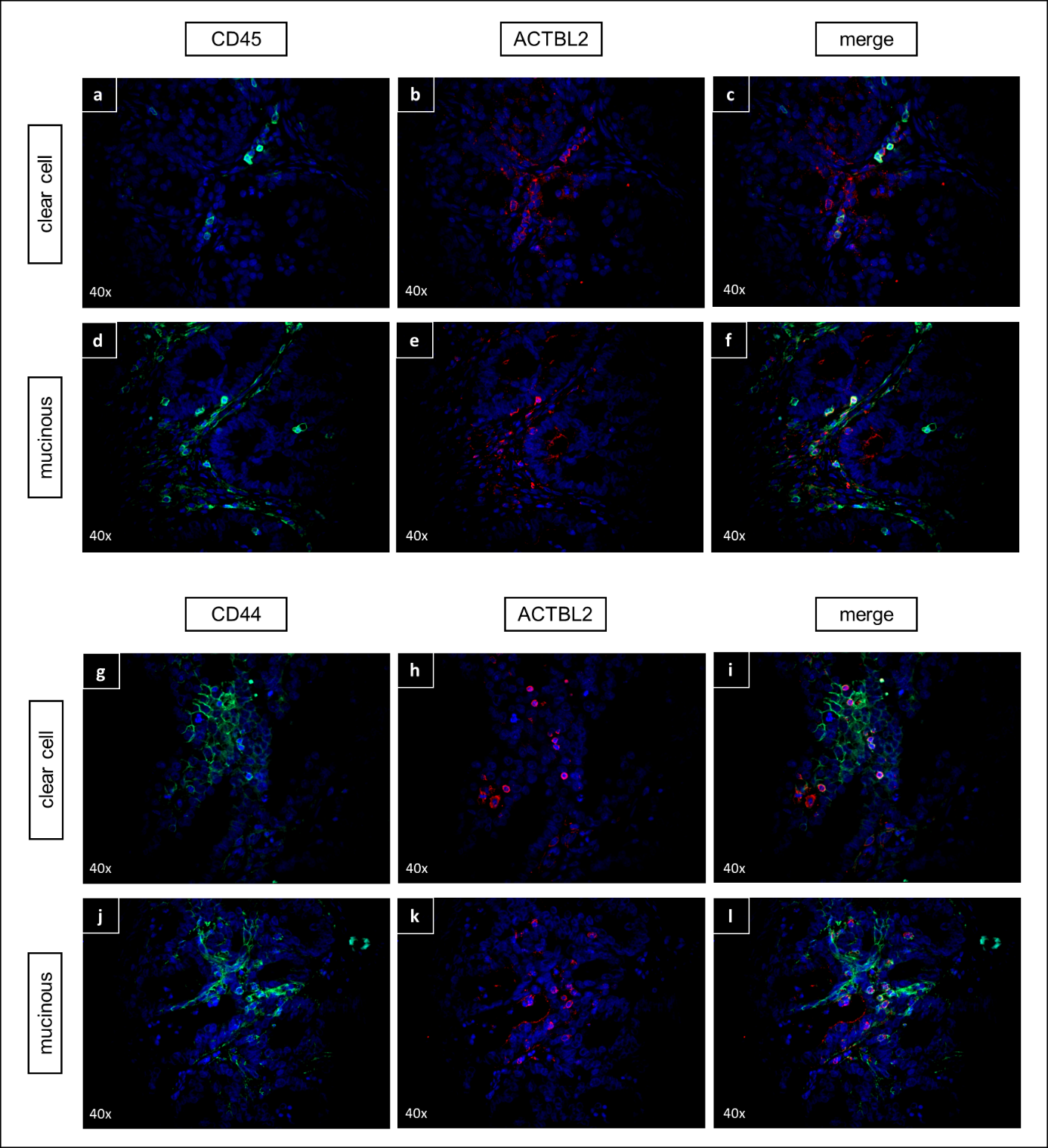


**Figure SX| Immunofluorescence double-staining with anti-ACTBL2, anti-CD45 and anti-CD44 antibodies. (a-f)** Representative staining results of EOC tissue of clear cell (a-c) and mucinous (d-f) histology, demonstrating an ACTBL2 expression in CD45-positive tumor-infiltrating leukocytes. **(g-l)** Exemplary photographs of clear cell (g-i) and mucinous (j-l) carcinoma, showing a co-expression of ACTBL2 and membrane-bound CD44 as a marker for activated TILs (40x magnification).


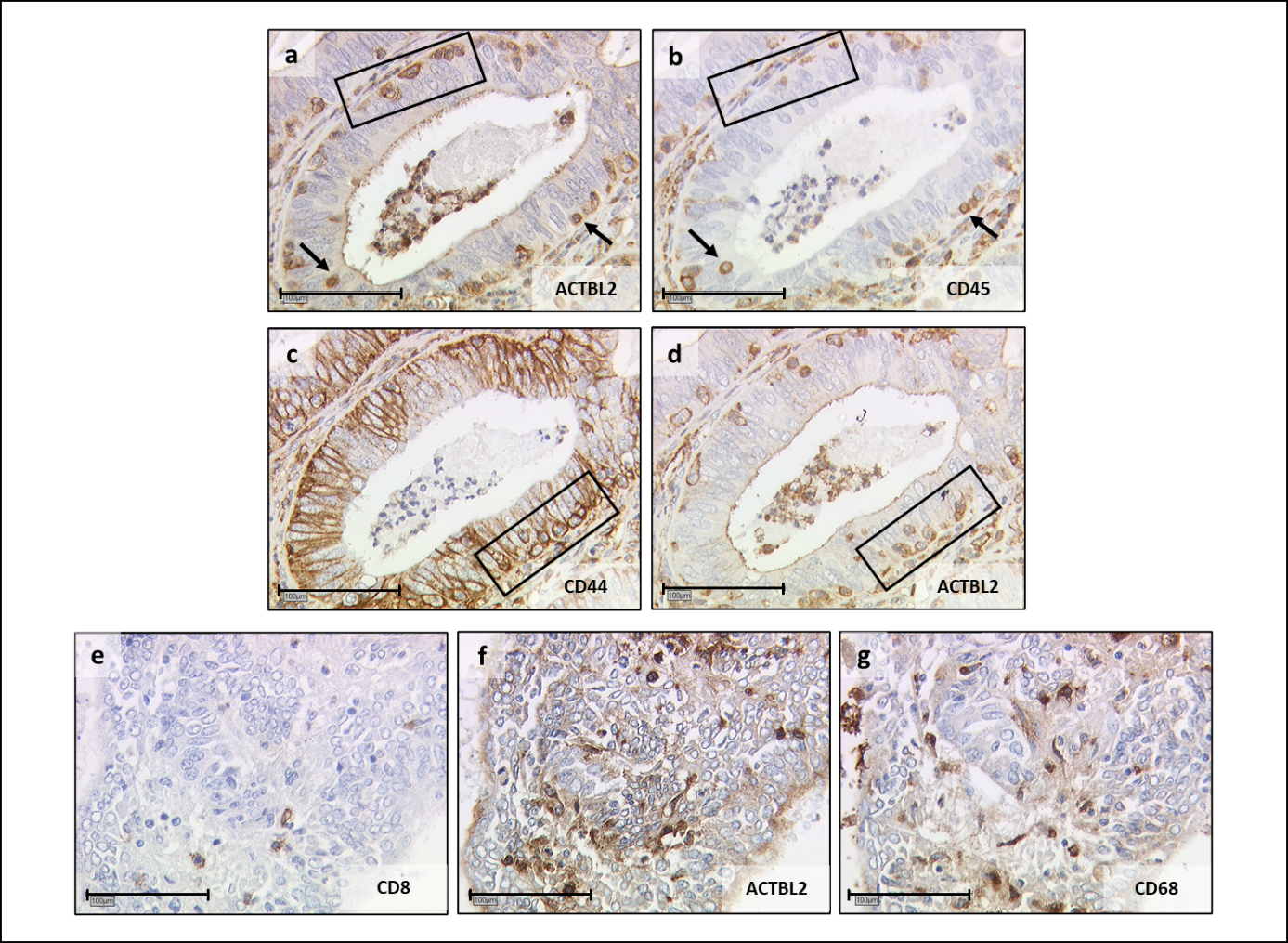


**Figure SX| Serial staining of mucinous carcinoma tissue for leukocyte subtyping. (a-d)** Exemplary photographs of consecutive mucinous carcinoma tissue slices after immunohistochemical staining of ACTBL2 (a and d), CD45 (b) and CD44 (c), identifying ACTBL2-overexpressing cells as tumor-infiltrating CD44-positive leukocytes. **(e-g)** Representative pictures of another mucinous carcinoma series, analyzing the presence of CD8-positive cytotoxic T-Cells (e) and CD68-positive macrophages (g) for a subtyping of ACTBL2-positive TILs (f). Identical cells between the pictures were marked by rectangles and arrows (25x magnification, scale bar=100µm).


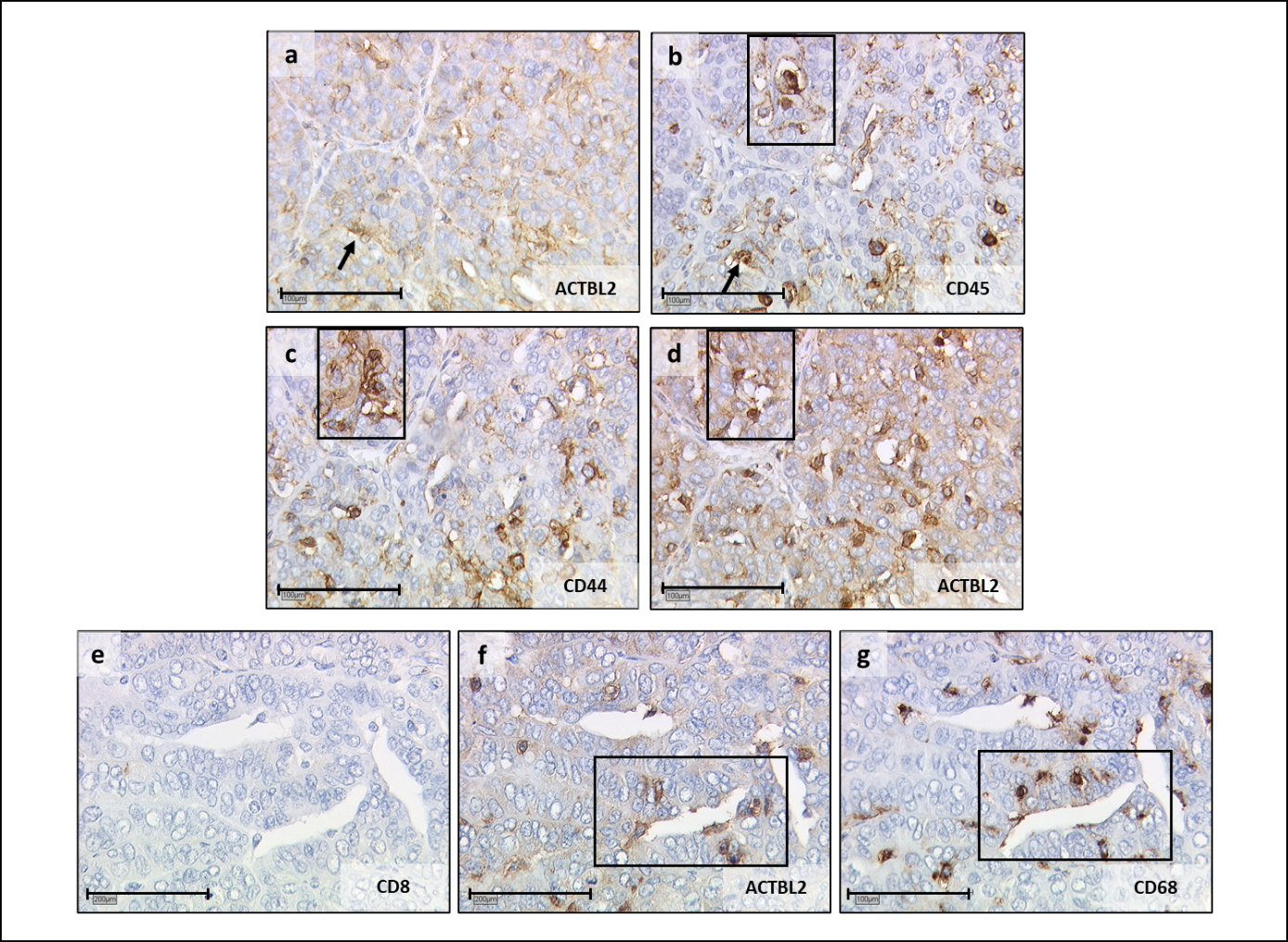


**Figure SX| Serial staining of serous carcinoma tissue for leukocyte subtyping. (a-d)** Exemplary photographs of consecutive serous carcinoma tissue slices after immunohistochemical staining of ACTBL2 (a and d), CD45 (b) and CD44 (c), identifying ACTBL2-overexpressing cells as tumor-infiltrating CD44-positive leukocytes. **(e-g)** Representative pictures of another serous carcinoma series, examining the co-expression of CD8 (e) or CD68 (g) of ACTBL2-positive TILs (f). Identical cells between the pictures were marked by rectangles and arrows (25x magnification, scale bar=100µm).


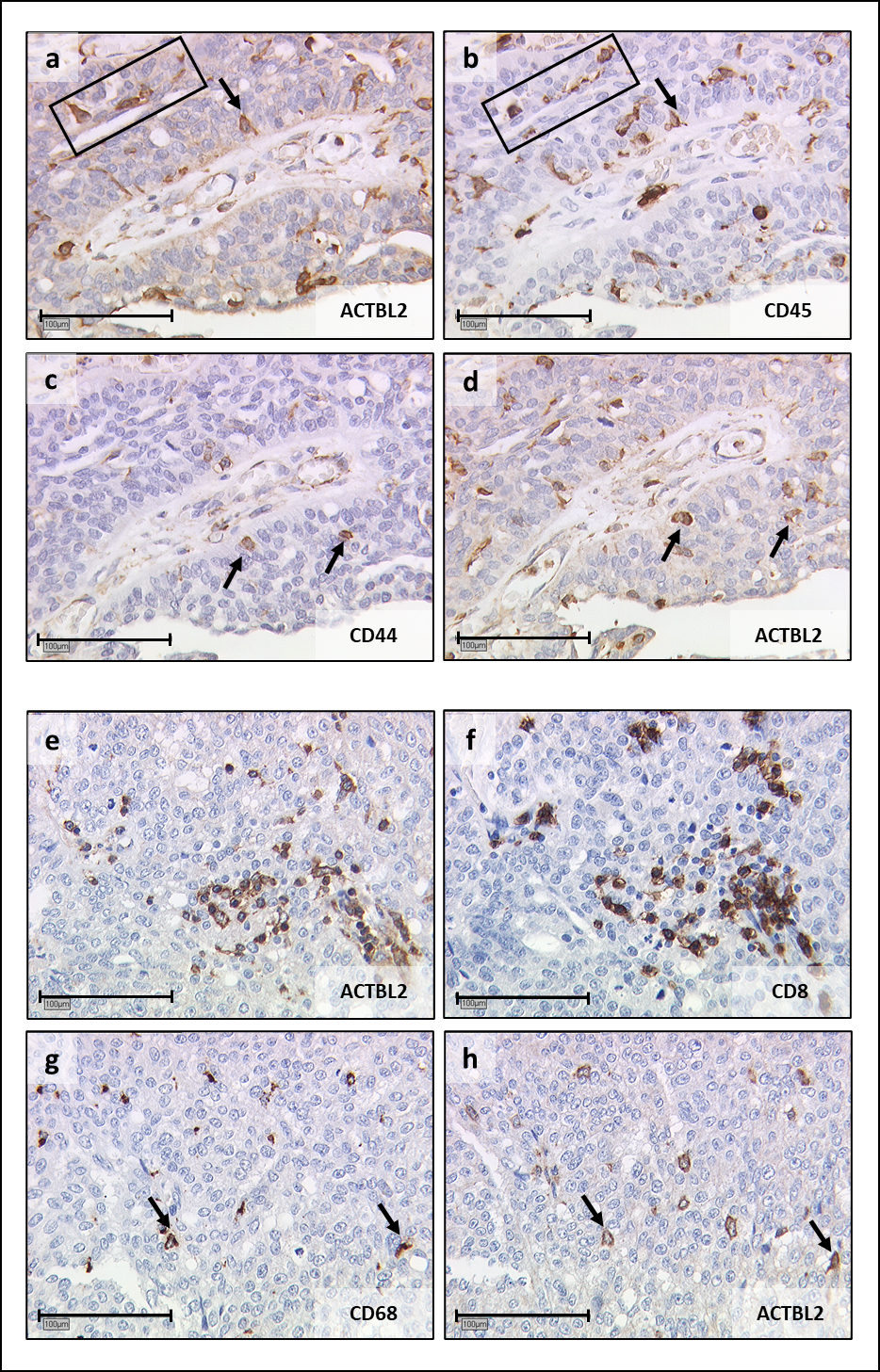


**Figure 4| Serial staining of endometrioid ovarian cancer for leukocyte subtyping. (a-d)** Representative photographs of an immunohistochemical staining series of endometrioid ovarian cancer, hinting at the co-expression of CD45 (b) and CD44 (c) by ACTBL2-positive (a and d) immune cells. **(e-h)** Exemplary pictures of two further series, identifying ACTBL2-overexpressing leukocytes (e and h) as CD8-positive (f) and CD68-expressing (g) immune cells. Identical cells between the pictures were marked by rectangles and arrows (25x magnification, scale bar=100µm).
